# Novozym 435-Catalyzed Synthesis of Well-Defined Hyperbranched Aliphatic Poly(β-thioether ester)

**DOI:** 10.3390/molecules25030687

**Published:** 2020-02-06

**Authors:** Wan-Xia Wu, Zi Liu

**Affiliations:** College of Pharmacy and Biological Engineering, Chengdu University, Chengdu 610106, China; CDUliuzi@163.com

**Keywords:** Novozym 435, poly(β-thioether ester), hyperbranched, acid degradation, oxidation response

## Abstract

A series of new hyperbranched aliphatic poly(β-thioether ester)s were prepared by the enzymatic ring-opening polycondensation of 1,4-oxathiepan-7-one (OTO) and AB_2_/ABB’ comonomer with acid-labile β-thiopropionate groups. Two kinds of comonomers, methyl 3-((3-hydroxy-2-(hydroxymethyl)propyl)thio)propanoate (HHTP) and methyl 3-((2,3-dihydroxypropyl)thio)propanoate (DHTP), with different primary alcohols and secondary alcohols, were synthesized by thiol–ene click chemistry and thiol-ene Michael addition, respectively. Immobilized lipase B from *Candida antarctica* (CALB), Novozym 435, was used as the catalyst. The random copolymers were characterized by ^1^H-NMR, ^13^C-NMR, GPC, TGA, and DSC. All branched copolyesters had high molecular weights over 15,000 Da with narrow polydispersities in the range of 1.75–2.01 and were amorphous polymers. Their degradation properties under acidic conditions were also studied in vitro. The polymeric nanoparticles of hyperbranched poly(β-thioether ester)s were successfully obtained and showed good oxidation-responsive properties, indicating their potential for biomedical applications.

## 1. Introduction

Over the past few decades, hyperbranched polymers (HBPs) have been widely studied and applied in the fields of biomedical materials, coatings, additives, nanotechnology, and supramolecular science [1,2,3]. HBPs are three-dimensional structured macromolecules, and have several unique physical/chemical properties, such as a low viscosity, good solubility and multi-functionalities [4,5,6]. To date, many HBPs with different chemical architectures, such as polyethers [7], polyethyleneimine [8], polyamides [9], polyurethanes [10,11], polyesters [12,13], and polycarbonates [14], have been described, and some of them have been commercialized, including Lupasol, Hybranes, Boltorn and so on [15,16]. Among them, hyperbranched aliphatic polyesters have attracted wide attention for drug delivery systems and tissue engineering scaffolds due to their favorable biodegradability and biocompatibility [13,17,18,19]. However, traditional hyperbranched aliphatic polyesters based on poly(glycolide) (PGA), poly(lactide) (PLA), and poly(ε-caprolactone) (PCL) usually exhibit slow degradation rates and lack functional groups on the polymer backbone, which limits their use in some demanding biomedical applications [20,21,22,23].

Poly(β-thioether ester)s (PTEs)—one of the sulfur-containing functional polyesters—have gradually attracted attention for drug delivery systems because of their unique acid-degradable and oxidation-responsive properties [24,25,26]. The β-thiopropionate groups in the polymer backbone can be selectively hydrolyzed under mild acidic conditions (pH ~5.5) at a very slow rate [24,27], and the hydrophobic thioethers are known to readily oxidize to more hydrophilic sulfoxides or sulfones when exposed to reactive oxygen species (ROS), such as hypochlorous acid (HClO) or hydrogen peroxides (H_2_O_2_) [28,29]. A large number of studies have shown that cancer cells have a slightly acidic environment with lower pH [30] and higher ROS levels than normal cells [31]. It is highly desirable to develop pH/ROS-responsive polymeric materials for the site-specific delivery of therapeutic agents. Poly(β-thioether ester)s are usually prepared by thiol-ene step growth polymerization from dithiols and diacrylate monomers. For example, Junkers and co-workers prepared linear poly(β-thioether ester)s [32] and cross-linked poly(β-thioether ester) networks [33] by amine-catalyzed thiol-ene click polymerization. Pu and co-workers developed a kind of AB-type amphiphilic diblock copolymer—methoxy poly(ethylene glycol)-*block*-poly(ester-thioether)—as a drug vehicle, by thiol-Michael addition polymerization [34]. Ghosh and co-workers synthesized an ABA-type amphiphilic triblock copolymer with acid-labile poly(β-thioether ester) as the hydrophobic segment by using a sequential thiol-acrylate Michael addition reaction [24]. Chen and co-workers reported a thermal and oxidation dual responsive poly(ethylene glycol) (PEG)-based poly(β-thioether ester) by the thiol–ene polymerization of PEG diacrylate and 1,2-ethanedithiol for ROS-triggered drug release [35]. Oh and co-workers demonstrated dual enzyme/oxidation-responsive poly(β-thioether ester)-based nanoparticles with controlled sizes for drug delivery [26]. Nevertheless, most of the reported poly(β-thioether ester)s are linear or network polymers, and very little attention has been dedicated to hyperbranched poly(β-thioether ester)s. Wang and co-workers reported the one-pot synthesis of amphiphilic hyperbranched poly(β-thioether ester)s (PPHD-PK) conjugated with cytotoxic peptide and poly(ethylene glycol) (PEG) based on a thiol-acrylate Michael addition reaction [36]. Ritter and co-workers prepared hyperbranched poly(β-thioether ester)s by the ring-opening polymerization of AB_2_-type lactones with a free hydroxyl group in the presence of Sn(Oct)_2_ as a catalyst at 110 °C with argon [37]. However, both thiol-ene step growth polymerization and metal-catalyzed polymerization often use toxic chemical catalysts and require inert gas protection. Additionally, the temperature of metal-catalyzed polymerization is quite high.

Lipase-catalyzed polymerization provides a new way to synthesize hyperbranched polymer materials [38,39]. It has the advantages of high catalytic activity, a high selectivity, a good tolerance of functional groups, mild reaction conditions, and the application of green biocatalysts [40]. Among lipases, *Candida antarctica* lipase B (CALB) has attracted great attention in polymer synthesis [41]. CALB can catalyze the hydrolysis of fatty acid esters in the original environment, while it can also catalyze the esterification reaction in an anhydrous medium, making CALB a widely used lipase in polymer chemistry [42]. Studies have shown that CALB-catalyzed esterification is more selective for primary hydroxyl groups than secondary hydroxyl groups [43,44]. Besides, the immobilization technique is a powerful tool for solving the problems of the reuse and stability of free lipase [45,46]. Novozym 435 is an immobilized preparation of CALB supplied by Novozymes, which has been extensively utilized for synthesizing polyester architectures [47,48]. Its support is a macroporous acrylic polymer resin (Lewatit VP OC 1600), where CALB is adsorbed by interfacial activation [47]. Frey and co-workers synthesized hyperbranched aliphatic copolyesters from ε-caprolactone and 2,2-bis(hydroxymethyl)butyric acid, catalyzed by immobilized CALB under mild conditions [49]. Gross and co-workers reported branched polymers produced by the CALB-catalyzed copolymerization of trimethylolpropane, 1,8-octanediol, and adipic acid [50], and they also described an enzyme-catalyzed route to polycarbonate polyols from diethyl carbonate, 1,8-octanediol, and tris(hydroxymethyl)ethane [51]. Wu and co-workers prepared a series of novel poly(amine-ester)-type HBPs through CALB-catalyzed polycondensation between triethanolamine (TEOA) and diesters [52]. Recently, Ronda and co-workers demonstrated renewable hyperbranched poly(10,11-dihydroxyundecanoic acid) by the self-polycondensation of 10,11-dihydroxyundecanoic acid using CALB as a catalyst [53]. However, the biocatalytic synthesis of hyperbranched poly(β-thioether ester) has never been studied. Only linear poly(β-thioether ester)s obtained by enzymatic polymerization have been reported [54,55,56,57,58]. For example, Yu and co-workers prepared linear poly(β-thioether ester) and its amphiphilic block copolymers through enzyme-catalyzed polycondensation using CALB [55,56,57]. Galià and co-workers reported poly(β-thioether ester)s-containing alkyl groups in the side chain based on a hydroxyester monomer sourced from castor oil [58]. Based on these previous studies, the development of a biocatalysis method for efficiently synthesizing hyperbranched poly(β-thioether ester) is highly desirable.

In this work, two kinds of hyperbranched aliphatic poly(β-thioether ester)s were synthesized by the Novozym 435-catalyzed ring-opening polycondensation of 1,4-oxathiepan-7-one (OTO) with different AB2/ABB′ comonomers. All the monomers contained a β-thiopropionate group. The acid-degradable behavior of hyperbranched poly(β-thioether ester) was studied. Furthermore, the polymeric nanoparticles of hyperbranched poly(β-thioether ester)s were prepared and their oxidation-responsive properties were further examined.

## 2. Results and Discussion

### 2.1. Synthesis of AB_2_/ABB’ Monomers

**Scheme 1:**
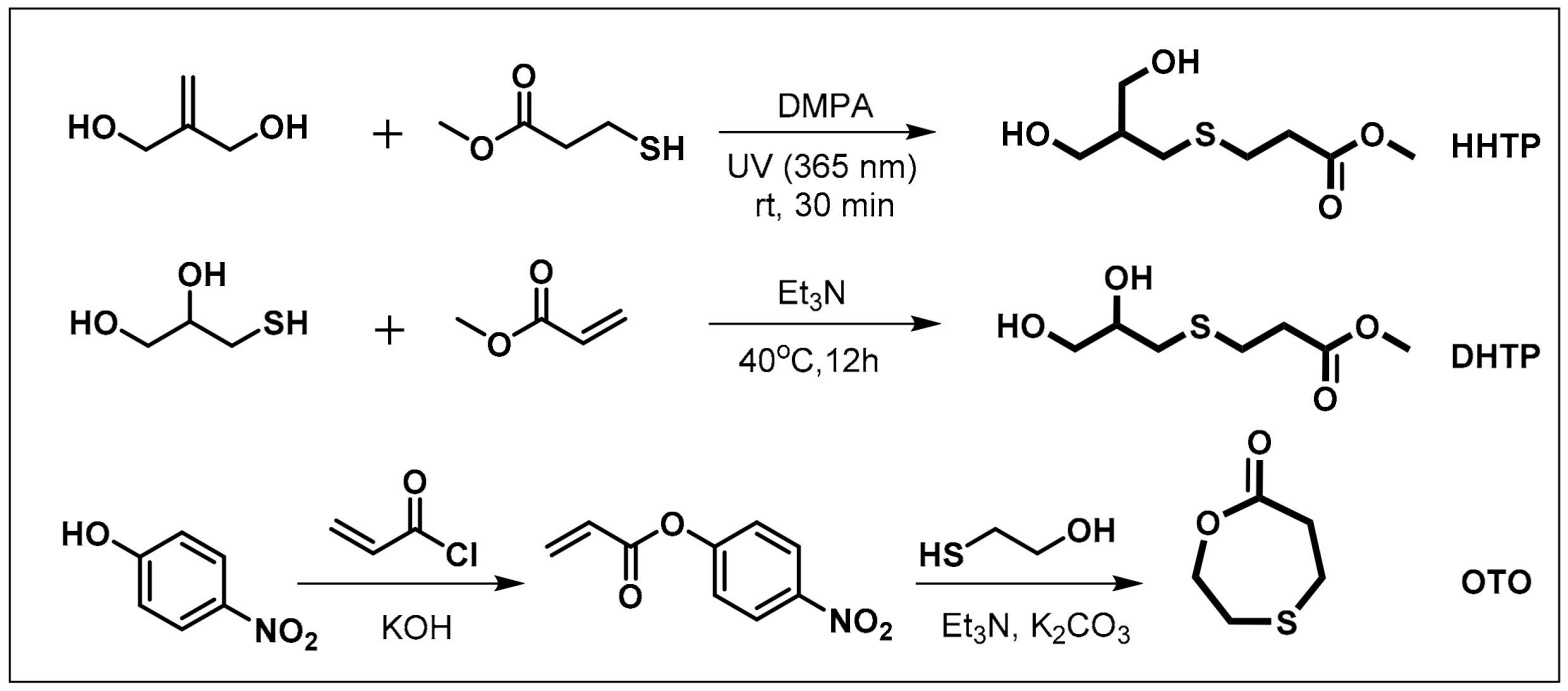
Synthetic route of monomers.

Scheme 1 shows the route of preparation of three monomers containing a β-thiopropionate group. The AB_2_ monomer—3-((3-hydroxy-2-(hydroxymethyl)propyl)thio)propanoate (HHTP)—was synthesized by thiol–ene click chemistry. The reaction of 2-methylenepropane-1,3-diol and 3-mercaptopropanoate was carried out under UV irradiation (365 nm), with a catalytic amount of DMAP, to produce the HHTP monomer in an 86% yield. The ABB’ monomer—methyl 3-((2,3-dihydroxypropyl)thio)propanoate (DHTP)—was obtained in a 66% yield through thiol-ene Michael addition from 3-mercaptopropane-1,2-diol and methyl acrylate, using triethylamine as the base catalyst. The lactone monomer—1,4-oxathiepan-7-one (OTO)—was prepared from 2-mercaptoethanol and 4-nitrophenyl acrylate, using the method reported by Li and co-workers [59]. Their formations were confirmed by NMR spectra.

### 2.2. Novozym 435-Catalyzed Synthesis of Hyperbranched Poly(β-thioether ester)s

**Scheme 2:**
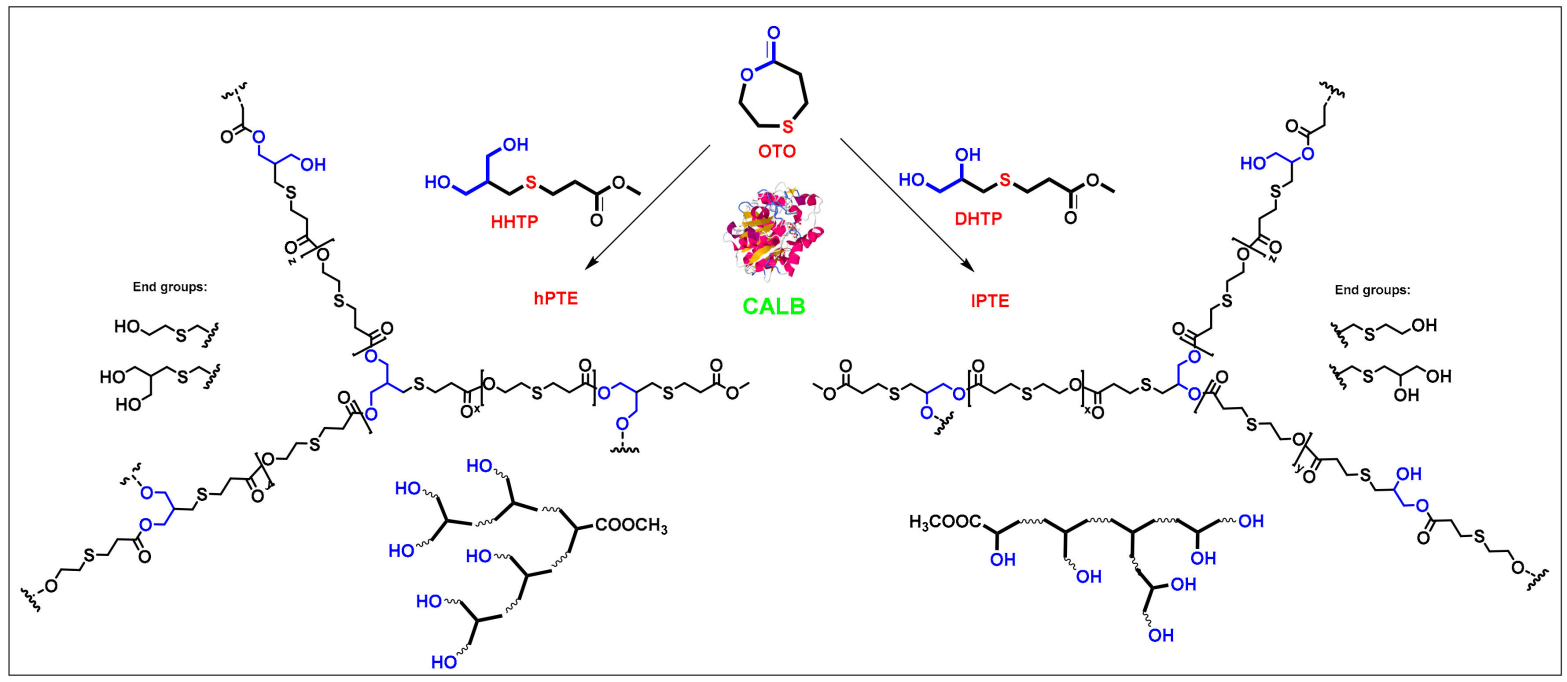
Novozym 435-catalyzed synthesis of hyperbranched poly(β-thioether ester)s.

**Figure 1:**
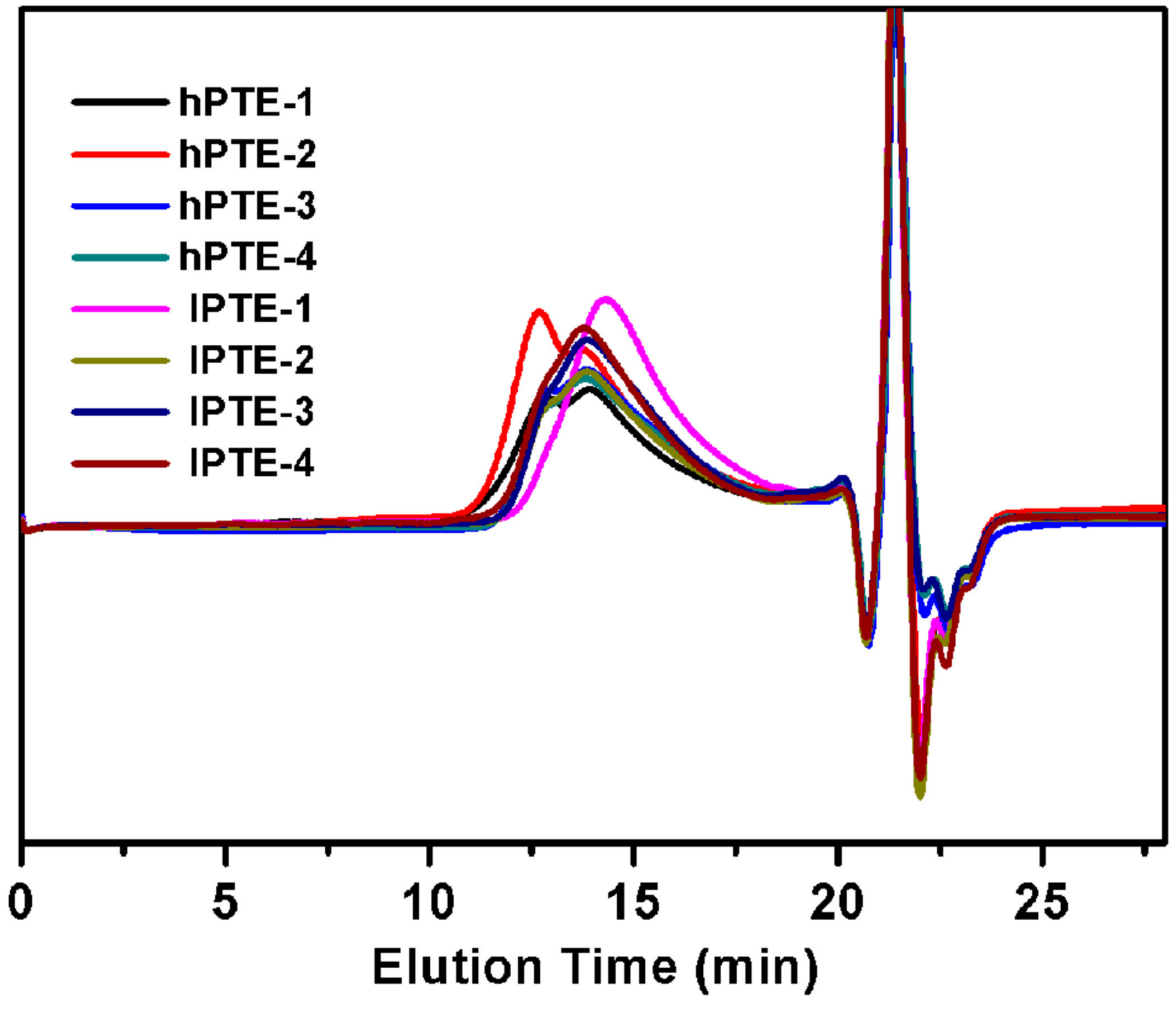
GPC traces of hPTE and lPTE copolymers.

As shown in Scheme 2, two types of hyperbranched poly(β-thioether ester)s—hPTE and lPTE—were prepared by Novozym 435-catalyzed ring-opening polycondensation from HHTP or DHTP with OTO. The reactions were carried out in diphenyl ether at 90 °C for 48 h with 10 wt% Novozym-435 using different monomer feeds. The molar feed ratio of HHTP or DHTP and OTO was varied from 1:3 to 1:49. All the copolymers had a high yield over 84%. GPC traces of the copolyesters are provided in Figure 1 and the obtained molecular weights are listed in Table 1. The Mn values of hPTE copolymers were in the range of 19,400 to 30,200 Da and their PDI values ranged from 1.78 to 2.01. Additionally, the Mn values of lPTE copolymers ranged from 15,300 to 21,700 Da and they also had narrow PDI values between 1.75 and 1.83.

### 2.3. Structural Characterization of Hyperbranched Poly(β-thioether ester)s

**Figure 2:**
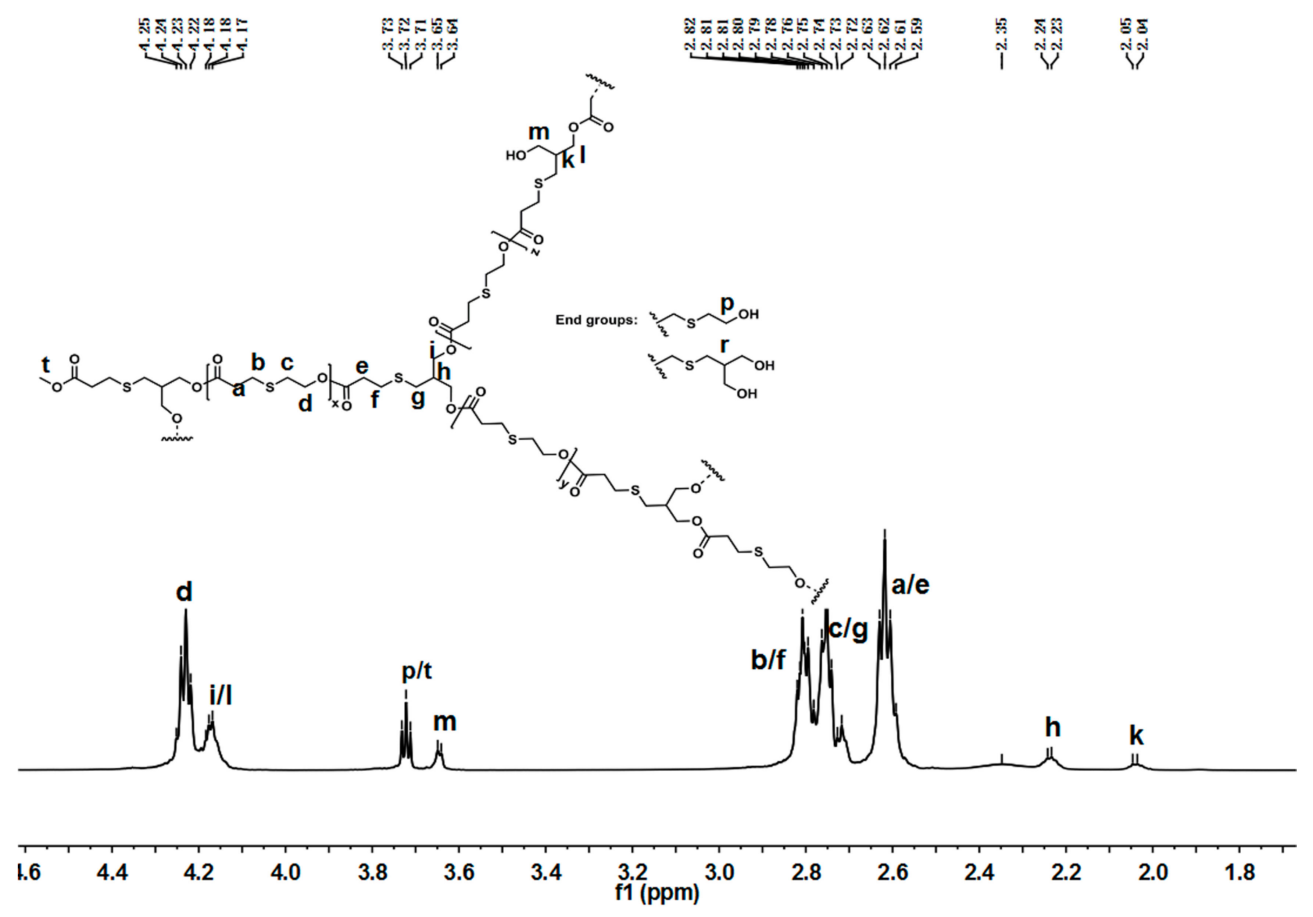
^1^H-NMR of hPTE-1 in CDCl_3_.

The chemical structure of hyperbranched poly(β-thioether ester)s was analyzed by NMR spectra. Figure 2 presents the ^1^H-NMR spectra of hPTE-1. As shown in Figure 2, the signals of OTO monomer at 4.57, 3.13, and 2.93 ppm disappeared, and new signals at 4.24, 2.81, and 2.75 ppm ascribed to the OTO unit appeared, indicating that ring-opening polymerization of OTO occurred. In addition, the disappearance of HHTP signals at 3.80 ppm and the appearance of new signals at 4.18 ppm confirmed the successful incorporation of HHTP into the polymer chains. There were three types of structural units, including linear, dendritic, and terminal units, in hyperbranched poly(β-thioether ester)s. For hPTE-1, the signal of the dendritic unit ascribed to the methenyl proton (C*H*CH_2_OOC) appeared at 2.23 ppm (*h*), and the methenyl signal of the linear unit appeared at 2.04 ppm (*k*). The methenyl signal of the terminal unit (*r*) from HHTP was not found in Figure 2. The signal ascribed to the methylene protons (C*H*_2_OH) of the terminal unit appeared at 3.72 ppm (*p*), which came from the OTO monomer. Moreover, the signal of the methylene protons (C*H*_2_OH) of the linear unit from HHTP appeared at 3.65 ppm (*m*). The proton signals at 4.24 and 3.72 ppm ascribed to the OTO unit and the proton signals at 4.18 and 3.65 ppm ascribed to the HTP unit were used to calculate the ratio of HTP versus OTO units in hPTE copolymers. The results are listed in Table 1. It was seen that the actual unit ratio was close to the feed ratio of monomers. The ^13^C-NMR spectra of hPTE-1 in Figure S6 also confirmed the structure. The characteristic peaks at 40.51 and 37.64 ppm were ascribed to the methenyl carbon atoms from linear and dendritic units, respectively. No peak of the terminal unit from HHTP was observed.

**Figure 3:**
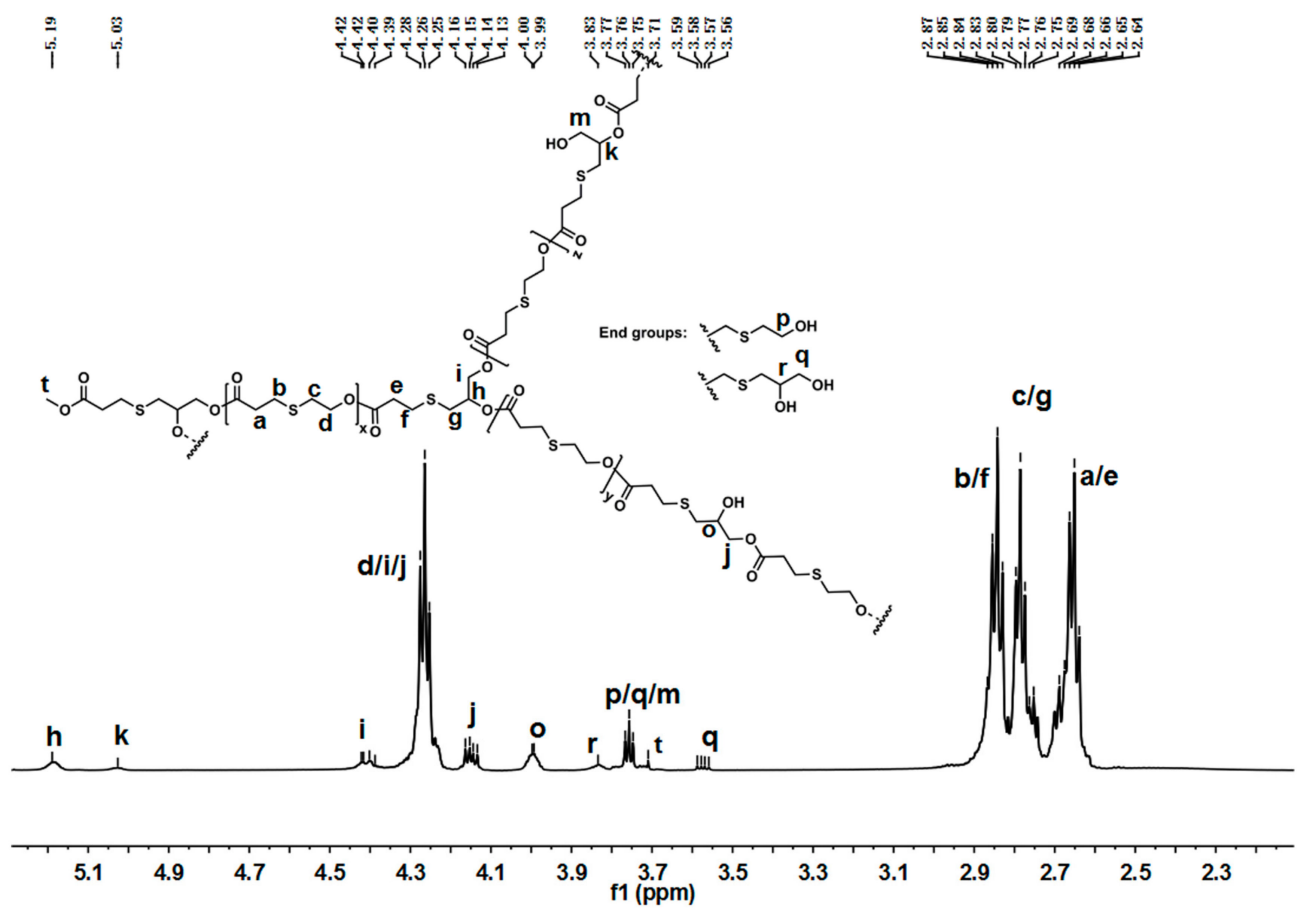
^1^H-NMR of lPTE-1 in CDCl_3_.

Figure 3 displays the ^1^H-NMR spectra of lPTE-1. The signals of the methenyl proton from the dendritic and terminal units appeared at 5.19 ppm (*h*) and 3.83 ppm (*r*), respectively. Unlike hPTE-1, lPTE-1 had two kinds of linear units from DHTP, which was due to the presence of two different hydroxyl groups in the ABB’ comonomer. The signals at 5.03 ppm (*k*) and 4.00 ppm (*o*) were ascribed to the HOCH_2_C*H*OOC proton and HOC*H*CH_2_OOC proton from the linear units, respectively. The intensity of the peak at 4.00 ppm (*o*) was significantly higher than that of the peak at 5.03 ppm (*k*), which may be due to the higher activity of the enzyme-catalyzed reaction on primary alcohols than secondary alcohols. The proton signals at 4.26 (*d*) ascribed to the OTO unit and the proton signals at 4.40 ppm (*i*) and 4.15 ppm (*j*) ascribed to the HTP unit were used to calculate the unit ratio in lPTE copolymers. The results in Table 1 showed that the composition of the copolymer could be controlled by changing the feed ratio. The ^13^C-NMR spectra of lPTE-1 are provided in Figure S7. The characteristic peaks at 74.18 ppm (*r*), 70.84 ppm (*h*), and 68.44 ppm (*o*) were attributed to the methenyl carbon atoms from terminal, dendritic, and linear units, respectively. Furthermore, the signals of the methylene carbon atoms of terminal units from OTO and DHTP appeared at 60.63 ppm (*p*) and 65.29 ppm (*q*), respectively. From the ^1^H-NMR spectra, the contents of linear AB_2_ or ABB’ units were calculated to be about 27.5% for hPTE-1 and 55.8% for lPTE-1 (Table 1). As shown in Table 1, the LHTP value of lPTE copolymers was higher than that of hPTE copolymers, suggesting that lPTE copolymers contained more linear structures. The degree of branching (DB) calculated according to a Frey definition [60]—DB = 2D/(2D + L)—was 0.348 for hPTE-1 and 0.131 for the lPTE-1 copolymer. The data are listed in Table 1. It was found that the degree of branching of hPTE copolymers was higher than that of lPTE copolymers, and the DB value decreased with the increase of the OTO monomer ratio. The above results showed the successful formation of the regulated hyperbranched structure for hPTE and lPTE-1 copolymers.

### 2.4. Thermal Characterization of Hyperbranched Poly(β-thioether ester)s

**Figure 4:**
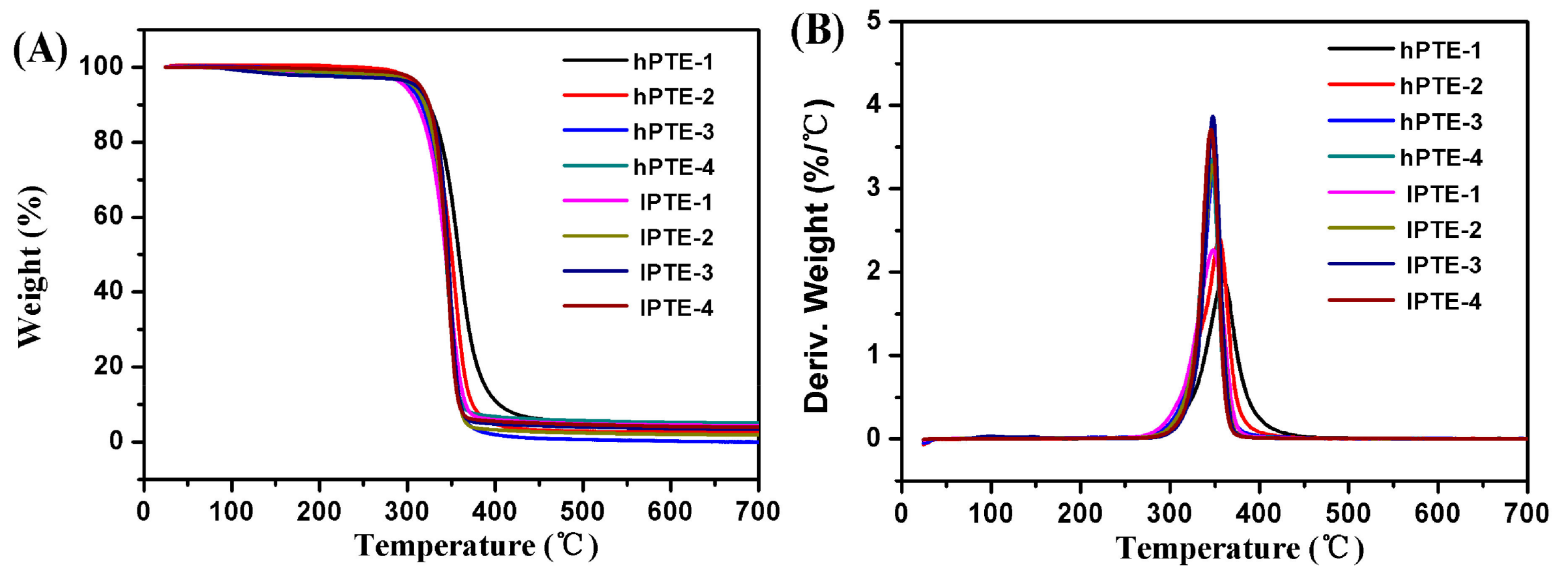
TGA curves (**A**) and derivative curves (**B**) of hPTE and lPTE copolymers at a heating rate of 10 °C min^−1^.

**Figure 5:**
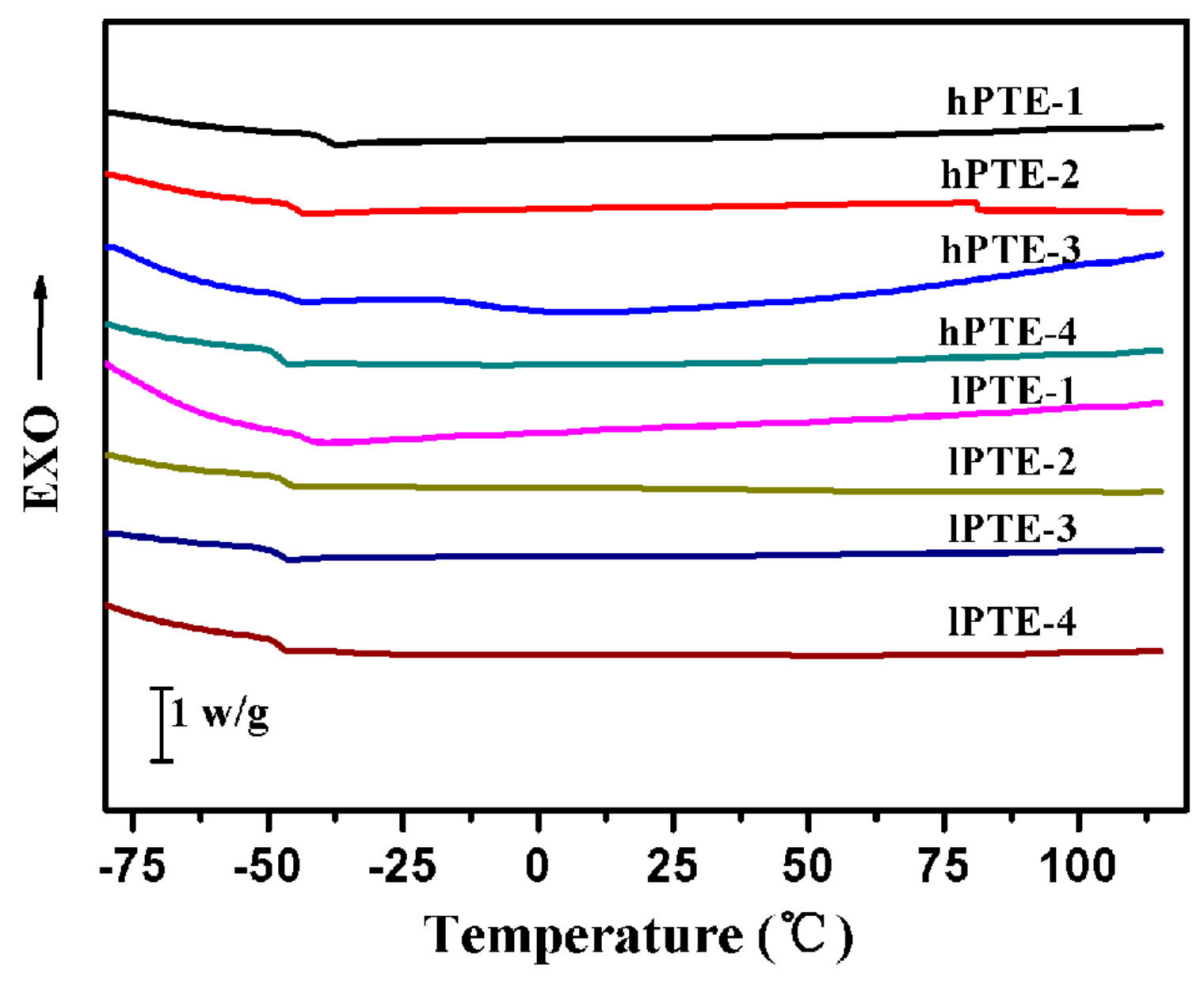
DSC curves of hPTE and lPTE copolymers at a heating rate of 10 °C min^−1^.

The thermal stabilities and behaviors of hPTE and lPTE copolymers were investigated by TGA and DSC. The TGA and TGA derivative curves are shown in Figure 4, and their data are provided in Table 2. As seen in Figure 4A, all the copolymers exhibited one degradation step and showed a good thermal stability. Additionally, all the remaining weight percentages (RW) at 700 °C were less than 5% (Table 2). For hPTE copolymers, the temperature at a 5% weight loss (T_5%_) was over 300 °C and the temperature for the maximum degradation rate (T_max_) was in the range of 346 to 359 °C. It could be found that the T_max_ value of hPTE copolymers decreased with an increasing OTO unit content. However, the T_max_ value of lPTE copolymers did not show the same trend, and their T_max_ values were close. The lPTE-1 copolymer had the minimum T_5%_ value among the copolymers. Compared to the linear poly(β-thioether ester)s reported by Yu et al. [55] and Li et al. [59], these hyperbranched poly(β-thioether ester)s exhibited a higher thermal stability. Figure 5 presents the DSC curves of hPTE and lPTE copolymers at a heating rate of 10 °C min^−1^, and the results are also presented in Table 2. The hPTE-1 copolymer showed a T_g_ of −43.4 °C, and lPTE-1 copolymer had a T_g_ of −38.8 °C. The T_g_ values of all the copolymers were higher than that of the linear poly(β-thioether ester)s (T_g_ = −49 °C) reported by Yu et al. [55], which may be due to the fact that hyperbranched polymers have greater steric hindrance than linear polymers, resulting in a reduced chain flexibility. From Table 2, it was found that the T_g_ values of the copolymers increased with the decrease of the molar fraction of AB_2_/ABB’ comonomer, which may be because of an increase in the degree of branching of the copolymers. Besides, no melting temperature (T_m_) and cold crystallization temperature (T_c_) were observed for these copolymers from DSC curves in the test temperature range (Figure 5 and Figure S8), indicating that the hPTE and lPTE copolymers had amorphous properties.

### 2.5. Acid-Degradation Study

**Figure 6:**
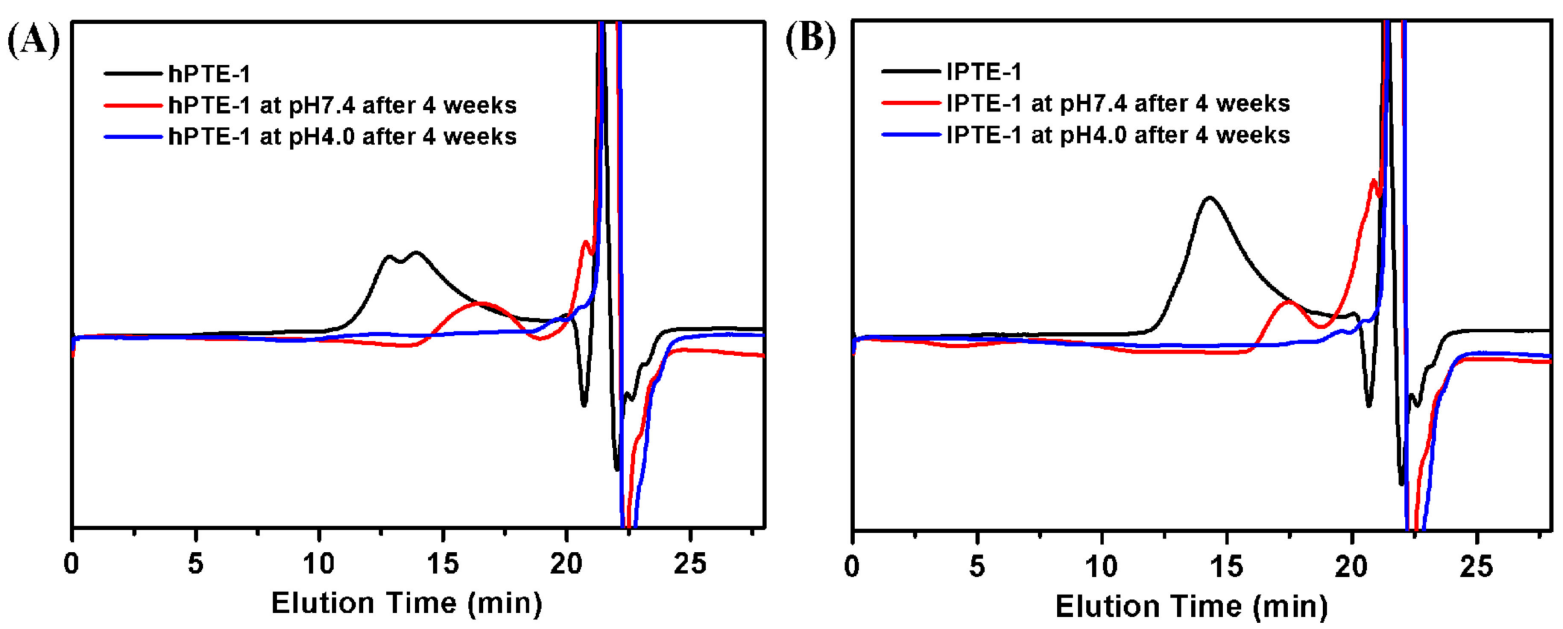
GPC traces of the degradation products of hPTE-1 (**A**) and lPTE-1 (**B**) at pH 7.4 and pH 4.0.

The degradation experiment of hyperbranched poly(β-thioether ester)s was carried out at 37 °C by in vitro hydrolysis at pH 7.4 and 4.0. Figure 6 shows the GPC curves for the degradation of hPTE-1 and lPTE-1. After 4 weeks in the buffer solution at pH 7.4, the Mn value of hPTE-1 decreased from 30,200 to 7300 Da and the PDI value changed from 1.78 to 1.45. However, the degradation rate of hPTE-1 at pH 4.0 was faster than at pH 7.4. After 4 weeks at pH 4.0, as shown in Figure 6A, the original copolymer peak of hPTE-1 disappeared and only peaks with small molecular weights appeared at positions with retention times of around 20 minutes. Moreover, the lPTE-1 copolymer exhibited similar degradation trends. After 4 weeks in the buffer solution at pH 7.4, the original peak of lPTE-1 was changed to two distinguishable peaks at low molecular weight positions (Figure 6B). The Mn value of the degradation product that appeared at positions with retention times of around 17 minutes was 4600 Da and the PDI value was 1.14. As shown in Figure 6B, the lPTE-1 copolymer could degrade rapidly after 4 weeks in the buffer solution at pH 4.0. The above experiments indicated that both hPTE-1 and lPTE-1 copolymers had acid-degradation characteristics.

### 2.6. Preparation and Characterization of Polymeric Nanoparticles

**Figure 7:**
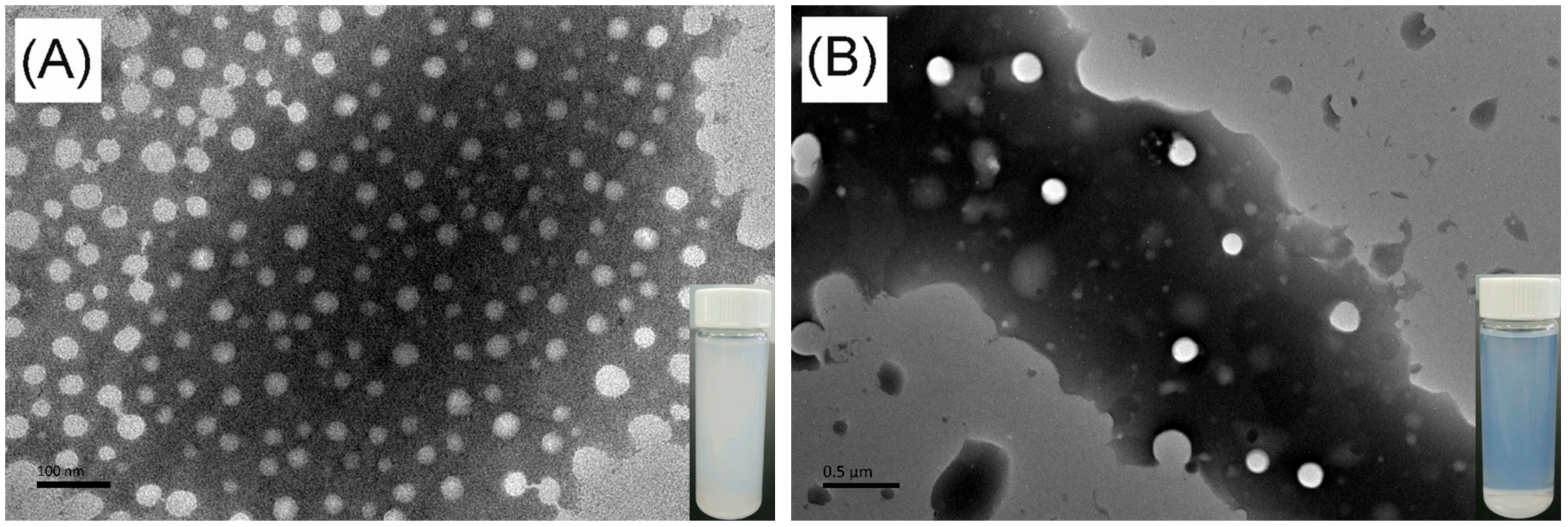
TEM images of hPTE-1 (**A**) and lPTE-1 (**B**) nanoparticles.

**Figure 8:**
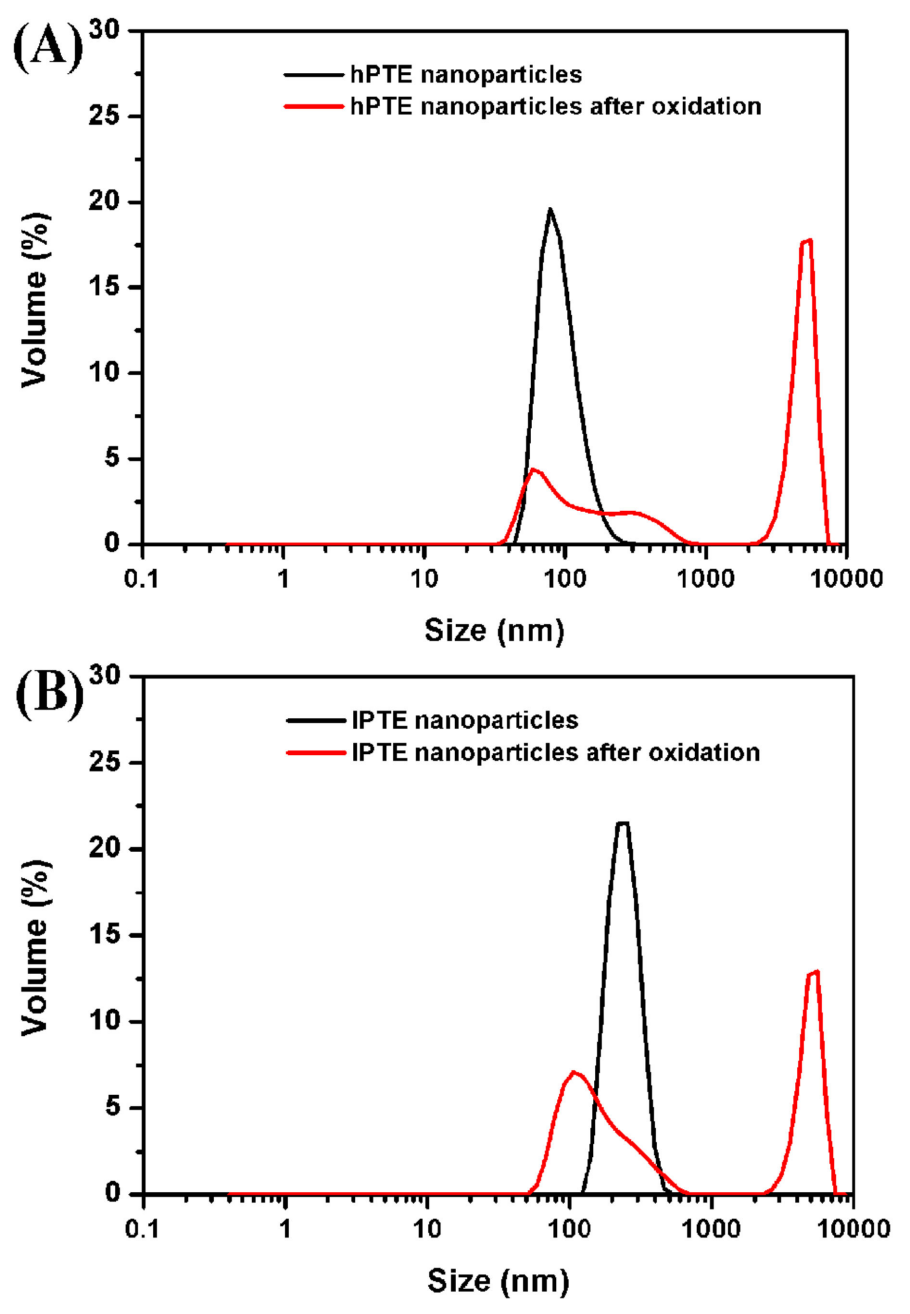
DLS plots of hPTE-1 (**A**) and lPTE-1 (**B**) nanoparticles treated with 3% (*w*/*v*) H_2_O_2_ at 25 °C for 4 h.

The polymeric nanoparticles of hyperbranched poly(β-thioether ester)s were prepared by a dialysis method [60]. The hPTE-1 or lPTE-1 copolymer was first dissolved in dioxane, and then deionized water was slowly added, followed by a dialysis process. As shown in Figure 7, the solution of hPTE-1 nanoparticles was milky white, while the solution of lPTE-1 nanoparticles was slightly bluish. It was found that the two kinds of polymeric nanoparticles exhibited a clear Tyndall scattering effect under red laser irradiation (Figure S9). The morphology of polymeric nanoparticles was investigated by transmission electron microscopy (TEM). As can be seen in the TEM images in Figure 7, both hPTE-1 and lPTE-1 nanoparticles were regularly spherical and well-dispersed. The size of hPTE-1 nanoparticles was smaller than that of lPTE-1 nanoparticles. Dynamic light scattering (DLS) measurements were also performed to confirm the sizes of polymeric nanoparticles. The DLS plots of polymeric nanoparticles are provided in Figure 8. The size of hPTE-1 nanoparticles was 94 ± 1 nm, with a narrow diameter distribution (PdI = 0.099 ± 0.002). However, the lPTE-1 nanoparticles showed a larger particle size of 248 ± 3 nm, with a PdI of 0.025 ± 0.012, which may be because of the formation of more loose aggregates resulting from the lower degree of branching of lPTE-1 than that of hPTE-1.

### 2.7. Oxidation-Responsive Study

**Figure 9:**
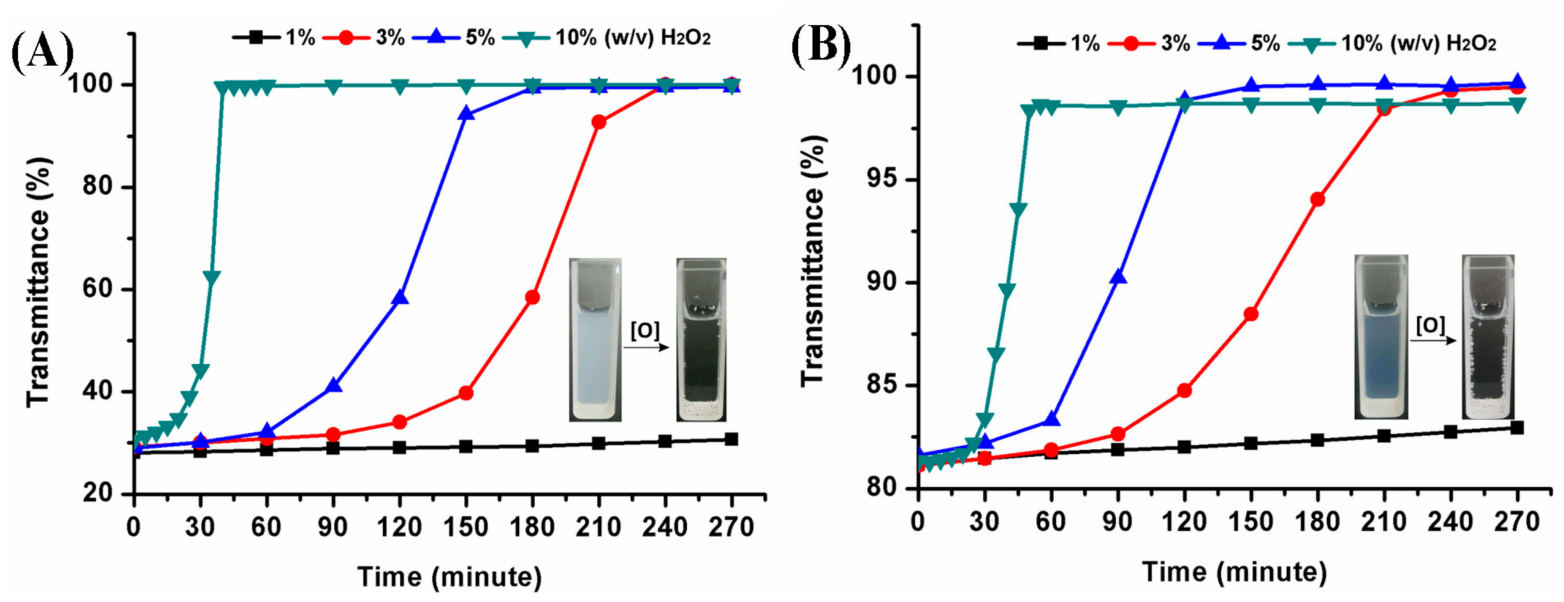
Turbidity measurements of hPTE-1 (**A**) and lPTE-1 (**B**) nanoparticles at different concentrations of H_2_O_2_ at 25 °C.

The oxidation-responsive behavior of hyperbranched poly(β-thioether ester)s was investigated by using hydrogen peroxide as a model oxidant. The dynamic oxidation process of the polymeric nanoparticles was monitored with UV. Figure 9 provides the turbidity measurements of hPTE-1 and lPTE-1 nanoparticles at different concentrations of H_2_O_2_. The original light transmittance of the hPTE-1 nanoparticle solution was lower than that of the lPTE-1 nanoparticle solution. As shown in Figure 9A, when the concentration of H_2_O_2_ was 1% (*w*/*v*), the transmittance of the hPTE-1 nanoparticle solution changed very slowly. However, a significant transition for the nanoparticle solution from turbid to transparent was observed after 4 h at 3% (*w*/*v*) H_2_O_2_, and the higher concentration of H_2_O_2_ accelerated the transmittance changes, which was due to the oxidation of the hydrophobic thioether groups to the hydrophilic sulfoxide or sulfone [29]. When the concentration of H_2_O_2_ reached 10% (*w*/*v*), the transmittance of the hPTE-1 nanoparticle solution increased up to 99.5% after 40 minutes. Similarly, the lPTE-1 nanoparticle solution gradually became transparent upon the addition of H_2_O_2_ (Figure 9B). The oxidation-responsive behavior was also studied by DLS. From Figure 8, it was found that the size of hPTE-1 and lPTE-1 nanoparticles significantly increased and was very dispersed after the oxidation of H_2_O_2_, demonstrating the disassociation of aggregates. These results exhibited that both hPTE-1 and lPTE-1 nanoparticles had good oxidation-responsive characteristics, which suggested their potential as a smart nanomaterial for drug delivery.

## 3. Materials and Methods

### 3.1. Materials

2-methylenepropane-1,3-diol (98%), 3-mercaptopropane-1,2-diol (98%), methyl 3-mercaptopropanoate (98%), triethylamine (99%), and 2,2-dimethoxy-1,2-diphenylethanone (DMPA) (98%) were purchased from Energy Chemical (Shanghai, China) and used as received. Methyl acrylate (99%) and diphenyl ether (99%) were purchased from Aladdin reagent (Shanghai, China) and used as received. Novozym 435 (Immobilized lipase B from *Candida antarctica*, CALB) was purchased from Novozymes (Bagsvaerd, Denmark) and used as received. All solvents and other chemicals were of analytical grade and were used without further purification. 1,4-oxathiepan-7-one (OTO) was synthesized according to a method presented in the literature [59].

### 3.2. Characterization

^1^H-NMR and ^13^C-NMR spectra were recorded with a JEOL-600M Spectrometer (JOEL Ltd., Tokyo, Japan), using CDCl_3_ as the solvent and TMS as an internal standard. The molecular weights and distributions of polyesters were measured by Gel permeation chromatography (GPC) (Waters Co., Milford, MA, USA) in THF in a Waters HPLC system (Waters Co., Milford, MA, USA) equipped with a 2414 refractive index (RI) detector (Waters Co., Milford, MA, USA) using polystyrene as the standard with a set of HT3 and HT4 at 45 °C. Thermogravimetric analysis (TGA) was measured with a TA Instruments TGA Q-500 thermogravimetric analyzer (TA Instrument, New Castle, DE, USA) under a nitrogen atmosphere at a heating rate of 10 °C min^−1^ from 25 to 700 °C. A differential scanning calorimetry (DSC) thermogram was produced using DSC Q200 apparatus (TA Instrument, New Castle, DE, USA) from TA instruments. The samples were heated quickly to 120 °C at 50 °C min^−1^ and maintained for 2 min to erase thermal history. Then, they were cooled to −80 °C at 10 °C min^−1^ and finally heated from −80 to 120 °C at 10 °C min^−1^ under a nitrogen atmosphere. UV-Vis absorption spectra were recorded on a Mapada UV-3100PC UV-visible spectrophotometer (Shanghai MAPADA Instruments, Shanghai, China). Dynamic light scattering (DLS) measurements were carried out at 25 °C using a Zetasizer Nano-ZS90 system (Malvern Instruments, Worcestershire, UK) equipped with a 633 nm He-Ne laser using backscattering detection with a fixed detector angle of 90°. Transmission electron microscopy (TEM, Hitachi H-600, Hitachi Co., Tokyo, Japan) studies were performed at an acceleration voltage of 100 kV. The samples were prepared by dropping the polymeric nanoparticle solution onto a copper grid, followed by negative staining with a 1 wt% aqueous solution of phosphotungstic acid.

### 3.3. Synthesis of Methyl 3-((3-hydroxy-2-(hydroxymethyl)propyl)thio)propanoate (HHTP)

2-methylenepropane-1,3-diol (1.89 g, 21.4 mmol), 3-mercaptopropanoate (2.58 g, 21.4 mmol), and 2,2-dimethoxy-2-phenylacetophenone (DMPA, 25 mg, 0.1 mmol), as the photoinitiator, were dissolved in 1 mL acetonitrile and transferred into a 25 mL quartz tube. The reaction mixture was irradiated with a 7 W lamp (λ = 365 nm) and stirred at room temperature for 30 minutes. The crude product was isolated by silica gel column chromatography with an eluent of ethyl acetate. The product was dried under vacuum to yield a colorless liquid (3.86 g, 86% yield).

^1^H-NMR (600 MHz, CDCl_3_), δ 3.80 (m, 4H, C*H*_2_OH), 3.71 (s, 3H, C*H*_3_), 2.83-2.63 (m, 4H, SC*H*_2_, O*H*), 2.66-2.63 (m, 4H, C*H*_2_CO, SC*H*_2_), 1.93 (m, 1H, C*H*). ^13^C-NMR (150 MHz, CDCl_3_), δ 172.68, 64.06, 51.95, 42.15, 34.54, 30.79, 27.54.

### 3.4. Synthesis of Methyl 3-((2,3-dihydroxypropyl)thio)propanoate (DHTP)

3-mercaptopropane-1,2-diol (5.41 g, 50 mmol) and methyl acrylate (4.30 g, 50 mmol) were placed in a 25 mL round bottom flask under magnetic stirring at room temperature. Triethylamine (0.1 mL, 0.7 mmol) was slowly added. The reaction mixture was stirred at 40 °C for 12 h. The crude product was isolated by silica gel column chromatography with an eluent consisting of hexane/ethyl acetate (1/2, *v*/*v*). The product was dried under vacuum to yield a pale yellow liquid (7.94 g, 66% yield).

^1^H-NMR (600 MHz, CDCl_3_), δ 3.83 (m, 1H, C*H*), 3.76–3.73 (m, 1H, C*H*_2_OH), 3.72 (s, 3H, C*H*_3_), 3.59–3.55 (m, 1H, C*H*_2_OH), 2.84 (t, 2H, SC*H*_2_), 2.77–2.72 (m, 1H, SC*H*_2_CH), 2.66–2.60 (m, 3H, SC*H*_2_CH, C*H*_2_CO), 2.40 (br, 2H, O*H*). ^13^C-NMR (150 MHz, CDCl_3_), δ 172.68, 70.88, 65.25, 51.99, 35.47, 34.57, 27.39.

### 3.5. Novozym 435-Catalyzed Polymerization of HHTP or DHTP with OTO Using Various Monomer Ratios

Mixtures contained HHTP or DHTP and OTO were placed into in a 25 mL round bottom flask. The molar ratios of HHTP or DHTP and OTO were 1:3, 1:9, 1:24, and 1:49. The total molar number of monomers was 5 mmol. Diphenyl ether (200 wt% vs. total monomer), as the solvent, was added to stir the mixture and Novozym 435 (10 wt% vs. total monomer) was transferred to the flask. The flask was stirred at 80 °C under reduced pressure, controlled by a Sciencetool C410 vacuum pump, for 48 h. The reaction was quenched with dichloromethane, and the enzyme was removed by filtration. The concentrated filtrate was washed with a large amount of hexane to extract and remove the diphenyl ether solvent and was subsequently precipitated three times using a mixture of dichloromethane and hexane (1:3, *v*/*v*). The products were dried under vacuum.

hPTE-1. ^1^H-NMR (600 MHz, CDCl_3_), δ 4.24 (m, SCH_2_C*H*_2_OOC), 4.18 (m, CHC*H*_2_OOC), 3.72 (m, C*H*_3_O, SCH_2_C*H*_2_OH end group), 3.65 (m, COOCH_2_CHC*H*_2_OH), 2.82–2.78 (m, SC*H*_2_CH_2_CO), 2.76–2.72 (m, SC*H*_2_CH_2_OOC, SC*H*_2_CH), 2.63–2.59 (m, SCH_2_C*H*_2_CO), 2.23 (m, C*H*CH_2_OOC dendritic unit), 2.04 (m, C*H*(CH_2_OH)CH_2_OOC linear unit). ^13^C-NMR (150 MHz, CDCl_3_), δ 172.01, 171.69, 171.51, 63.95, 63.70, 63.60, 63.47, 61.55, 60.67, 40.51, 37.64, 35.30, 34.76, 34.70, 34.57, 30.86,30.71, 30.52, 27.54, 27.12, 26.77, 26.64.

lPTE-1. ^1^H-NMR (600 MHz, CDCl_3_), δ 5.19 (m, COOCH_2_C*H*OOC dendritic unit), 5.03 (m, HOCH_2_C*H*OOC linear unit), 4.40 (m, COOC*H*_2_CHOOC), 4.26 (m, SCH_2_C*H*_2_OOC, COOC*H*_2_CHOOC, HOCHC*H*_2_OOC), 4.15 (m, HOCHC*H*_2_OOC), 4.00 (m, HOC*H*CH_2_OOC), 3.83 (m, C*H*CH_2_OH end group), 3.76 (m, HOC*H*_2_CHOOC, SCH_2_C*H*_2_OH end group, CHC*H*_2_OH end group), 3.71 (s, C*H*_3_O end group), 3.58 (m, CHC*H*_2_OH end group), 2.87–2.83 (m, SC*H*_2_CH_2_CO), 2.80–2.75 (m, SC*H*_2_CH_2_OOC, SC*H*_2_CH), 2.69–2.64 (m, SCH_2_C*H*_2_CO). ^13^C-NMR (150 MHz, CDCl_3_), δ 171.68, 171.51, 74.18, 70.84, 68.44, 67.16, 65.29, 63.88, 63.70, 63.60, 60.63, 35.94, 35.33, 34.71, 32.26, 30.53, 27.43, 27.13, 26.64.

### 3.6. Acid-Sensitive Degradation of hPTE-1 and lPTE-1 Copolymers

The acid-degradation of hyperbranched polyesters was investigated in vitro. In total, 10 mg of hPTE-1 or lPTE-1 was dissolved in 2 mL dioxane. Then, 8 mL phosphate (pH = 7.4, 0.05 M) or acetate (pH = 4.0, 0.05 M) buffered solutions was added to the dioxane polymer solution. The mixtures were processed in a shaking incubator at 37 °C. After 4 weeks, samples were taken and immediately placed in a refrigerator at −20 °C. The residual solution was removed by freeze drying. The molecular weights of the degradation products were measured by Gel permeation chromatography (GPC).

### 3.7. Preparation of hPTE-1 and lPTE-1 Nanoparticles

In total, 10 mg of hPTE-1 or lPTE-1 was dissolved in 5 mL of dioxane in a 25 mL round bottom flask. A total of 20 mL of deionized water was dropwise added to the polymer solution under magnetic stirring. After that, the solution was transferred to a dialysis membrane (MWCO 3500 Da) and dialyzed against deionized water for 3 days, with water exchange every 8 h, to remove the organic solvent. The samples were diluted with deionized water, filtered through a 0.45 µm Millipore filter with a polymeric nanoparticle solution concentration of 0.33 mg mL^−1^, and stored at 4 °C for further use. The size and morphology of the polymeric nanoparticles were determined by DLS and TEM, respectively.

### 3.8. Oxidation-Responsive Properties of hPTE-1 and lPTE-1 Nanoparticles

The oxidation behavior of the nanoparticles with different concentrations of H_2_O_2_ was studied by monitoring the transmittance changes in aqueous solution at 25 °C. Typically, 0.09, 0.27, 0.45, and 0.90 mL of 30 wt% H_2_O_2_ were added to 1.82 mL of the polymeric nanoparticle solution (0.33 mg mL^−1^), and the mixture was diluted with deionized water to 3 mL. The samples were reacted at 25 °C and the optical transmittance of the solution was measured by UV-Vis spectrometer detection of the change in transmittance (λ = 500 nm) at room temperature.

## 4. Conclusions

In summary, novel hyperbranched aliphatic poly(β-thioether ester)s were successfully synthesized by Novozym 435-catalyzed polymerization. The polymers demonstrated high molecular weights over 15,000 Da and relatively narrow polydispersities (PDI < 2.1). The degree of branching of copolymers from the HHTP comonomer was higher than that of copolymers from the DHTP comonomer, and the DB value could be regulated by changing the monomer ratio. All the hyperbranched polymers exhibited a high thermally stability, with a T_max_ value above 340 °C, and were amorphous polyesters, with a T_g_ value ranging from −38.8 to −47.8 °C. Both hPTE-1 and lPTE-1 copolymers revealed rapid degradation under acidic conditions. Their polymeric nanoparticles had uniform sizes and regular spherical structures. Additionally, an in vitro oxidation experiment indicated that these polymeric nanoparticles could be destroyed by the oxidation stimuli due to the oxidation of thioether groups in the polymer backbone. These types of hyperbranched poly(β-thioether ester)s have potential for biomedical applications in the future.

## Figures and Tables

**Table 1:** Effect of the monomer ratio on the copolymerization of methyl 3-((3-hydroxy-2-(hydroxymethyl)propyl)thio)propanoate (HHTP) or methyl 3-((2,3-dihydroxypropyl)thio)propanoate (DHTP) with 1,4-oxathiepan-7-one (OTO).

Name	HHTP or DHTP:OTO (feed ratio)	HHTP or DHTP:OTO ^a^ (unit ratio)	L_HTP_ (%) ^b^	DB ^c^	Yield (%)	Mn ^d^	PDI ^d^
hPTE-1	1:3	1:4	27.5	0.348	87	30200	1.78
hPTE-2	1:9	1:9	25.9	0.203	93	31000	1.89
hPTE-3	1:24	1:26	9.9	0.049	89	19400	2.01
hPTE-4	1:49	1:47	3.8	0.015	84	21100	1.84
lPTE-1	1:3	1:3	55.8	0.131	90	15300	1.75
lPTE-2	1:9	1:8	52.2	0.094	97	21700	1.76
lPTE-3	1:24	1:20	47.4	0.013	86	19700	1.81
lPTE-4	1:49	1:31	40.8	0.007	93	21400	1.83

^a^ Determined by ^1^H-NMR. ^b^ L_HTP_ means linear AB_2_ or ABB’ units as a percentage of total AB_2_ or ABB’ units in the polymers, calculated from ^1^H-NMR. ^c^ DB is the degree of branching, determined by ^1^H-NMR. ^d^ Determined by Gel permeation chromatography (GPC) against polystyrene standards.

**Table 2:** Thermal characterization of hyperbranched poly(β-thioether ester)s.

Name	T_5%_^a^ (°C)	T_max_^b^ (°C)	RW ^c^ (%)	Tm ^d^ (°C)	Tc ^d^ (°C)	Tg ^d^ (°C)
hPTE-1	310	359	4.8	-	-	−43.4
hPTE-2	310	355	2.6	-	-	−46.6
hPTE-3	304	349	0	-	-	−47.6
hPTE-4	307	346	5.0	-	-	−47.8
lPTE-1	297	348	4.3	-	-	−38.8
lPTE-2	311	351	1.8	-	-	−44.7
lPTE-3	311	349	3.3	-	-	−45.9
lPTE-4	316	347	3.9	-	-	−47.7

^a^ Temperature at 5% weight loss determined by TGA. ^b^ Temperature for the maximum degradation rate determined by TGA. ^c^ Remaining weight at 700 °C. ^d^ Determined d by DSC.

## Supplementary Materials

The following are available online: Figure S1: ^1^H-NMR spectra of OTO, Figure S2: ^1^H-NMR spectra of HHTP, Figure S3: ^13^C-NMR spectra of HHTP, Figure S4: ^1^H-NMR spectra of DHTP, Figure S5: ^13^C-NMR spectra of DHTP, Figure S6: ^13^C-NMR spectra of hPTE-1, Figure S7: ^13^C-NMR spectra of hPTE-1, Figure S8: DSC curves of hPTE and lPTE copolymers at a cooling rate of 10 °C min^−1^, Figure S9: Tyndall effect of hPTE and lPTE nanoparticle solution under red laser irradiation.

## References

[1] Caminade A., Yan D., Smith D.K. (2015). Dendrimers and hyperbranched polymers. Chem. Soc. Rev..

[2] Yates C.R., Hayes W. (2004). Synthesis and applications of hyperbranched polymers. Eur. Polym. J..

[3] Zhou Y., Huang W., Liu J., Zhu X., Yan D. (2010). Self-assembly of hyperbranched polymers and its biomedical applications. Adv. Mater..

[4] Kim Y.H. (1998). Hyperbranched polymers 10 years after. J. Polym. Sci. Part. A Polym. Chem..

[5] Voit B. (2005). Hyperbranched polymers-all problems solved after 15 years of research. J. Polym. Sci. Part. A Polym. Chem..

[6] Zheng Y., Li S., Weng Z., Gao C. (2015). Hyperbranched polymers: advances from synthesis to applications. Chem. Soc. Rev..

[7] Uhrich K.E., Hawker C.J., Frechet J.M.J., Turner S.R. (1992). One-pot synthesis of hyperbranched polyethers. Macromolecules.

[8] Zhang Q., Yu Q., Geng Y., Zhang J., Wu W., Wang G., Gu Z., Yu X. (2014). Ring-opening polymerization for hyperbranched polycationic gene delivery vectors with excellent serum tolerance. Acs Appl. Mater. Interfaces.

[9] Yang M., Wu Y., Liu Y., Qiu J., Liu C. (2019). A novel bio-based AB_2_ monomer for preparing hyperbranched polyamides derived from levulinic acid and furfurylamine. Polym. Chem..

[10] Spindler R., Frechet J.M.J. (1993). Synthesis and characterization of hyperbranched polyurethanes prepared from blocked isocyanate monomers by step-growth polymerization. Macromolecules.

[11] Rannard S.P., Davis N.J., Herbert I. (2004). Synthesis of water soluble hyperbranched polyurethanes using selective activation of AB_2_ monomers. Macromolecules.

[12] Hawker C.J., Lee R., Frechet J.M.J. (1991). One-step synthesis of hyperbranched dendritic polyesters. J. Am. Chem. Soc..

[13] Malmstroem E., Johansson M., Hult A. (1995). Hyperbranched aliphatic polyesters. Macromolecules.

[14] Tryznowski M., Tomczyk K., Fras Z., Gregorowicz J., Rokicki G., Wawrzyńska E., Parzuchowski P.G. (2012). Aliphatic hyperbranched polycarbonates: synthesis, characterization, and solubility in supercritical carbon dioxide. Macromolecules.

[15] Froehling P. (2004). Development of DSM’s hybrane^®^ hyperbranched polyesteramides. J. Polym. Sci. Part. A Polym. Chem..

[16] Aydogan C., Ciftci M., Yagci Y. (2018). Hyperbranched polymers by light-induced self-condensing vinyl polymerization. Macromol. Rapid Commun..

[17] Zou J., Shi W., Wang J., Bo J. (2005). Encapsulation and controlled release of a hydrophobic drug using a novel nanoparticle-forming hyperbranched polyester. Macromol. Biosci..

[18] Santra S., Kaittanis C., Perez J.M. (2010). Aliphatic hyperbranched polyester: a new building block in the construction of multifunctional nanoparticles and nanocomposites. Langmuir.

[19] Zhang H., Patel A., Gaharwar A.K., Mihaila S.M., Iviglia G., Mukundan S., Bae H., Yang H., Khademhosseini A. (2013). Hyperbranched polyester hydrogels with controlled drug release and cell adhesion properties. Biomacromolecules.

[20] Bednarek M. (2016). Branched aliphatic polyesters by ring-opening(co)polymerization. Prog. Polym. Sci..

[21] Fischer A.M., Frey H. (2010). Soluble hyperbranched poly(glycolide) copolymers. Macromolecules.

[22] Jikei M., Suzuki M., Itoh K., Matsumoto K., Saito Y., Kawaguchi S. (2012). Synthesis of hyperbranched poly(l-lactide)s by self-polycondensation of AB_2_ macromonomers and their structural characterization by light scattering measurements. Macromolecules.

[23] Smet M., Gottschalk C., Skaria S., Frey H. (2005). Aliphatic hyperbranched copolyesters by combination of ROP and AB_2_-polycondensation. Macromol. Chem. Phys..

[24] Dan K., Ghosh S. (2013). One-pot synthesis of an acid-labile amphiphilic triblock copolymer and its pH-responsive vesicular assembly. Angew. Chem. Int. Ed..

[25] Wu W., Yang X., Liu B., Deng Q., Xun M., Wang N., Yu X. (2016). Lipase-catalyzed synthesis of oxidation-responsive poly(ethylene glycol)-*b*-poly(β-thioether ester) amphiphilic block copolymers. RSC Adv..

[26] Hong S.H., Patel T., Ip S., Garg S., Oh J.K. (2018). Microfluidic assembly to synthesize dual enzyme/oxidation-responsive polyester-based nanoparticulates with controlled sizes for drug delivery. Langmuir.

[27] Oishi M., Nagasaki Y., Itaka K., Nishiyama N., Kataoka K. (2005). Lactosylated poly(ethylene glycol)-sirna conjugate through acid-labile β-thiopropionate linkage to construct ph-sensitive polyion complex micelles achieving enhanced gene silencing in hepatoma cells. J. Am. Chem. Soc..

[28] Lee S.H., Gupta M.K., Bang J.B., Bae H., Sung H. (2013). Current progress in reactive oxygen species (ROS)-responsive materials for biomedical applications. Adv. Healthcare Mater..

[29] Vo C.D., Kilcher G., Tirelli N. (2009). Polymers and sulfur: what are organic polysulfides good for? preparative strategies and biological applications. Macromol. Rapid Commun..

[30] Tannock I.F., Rotin D. (1989). Acid pH in tumors and its potential for therapeutic exploitation. Cancer Res..

[31] Yang B., Chen Y., Shi J. (2019). Reactive oxygen species (ROS)-based nanomedicine. Chem. Rev..

[32] Vandenbergh J., Ranieri K., Junkers T. (2012). Synthesis of (bio)-degradable poly(β-thioester)s via amine catalyzed thiol-ene click polymerization. Macromol. Chem. Phys..

[33] Vandenbergh J., Peeters M., Kretschmer T., Wagner P., Junkers T. (2014). Cross-linked degradable poly(β-thioester) networks via amine-catalyzed thiol-ene click polymerization. Polymer.

[34] Cheng F., Su T., Luo K., Pu Y., He B. (2019). The polymerization kinetics, oxidation-responsiveness, and in vitro anticancer efficacy of poly(ester-thioether)s. J. Mater. Chem. B.

[35] Xiao C., Ding J., Ma L., Yang C., Zhuang X., Chen X. (2015). Synthesis of thermal and oxidation dual responsive polymers for reactive oxygen species (ROS)-triggered drug release. Polym. Chem..

[36] Cheng D., Yang P., Cong Y., Liu F., Qiao Z., Wang H. (2017). One-pot synthesis of pH-responsive hyperbranched polymer-peptide conjugates with enhanced stability and loading efficiency for combined cancer therapy. Polym. Chem..

[37] Stöhr O., Ritter H. (2015). Hyperbranched polyesters based on hydroxyalkyl-lactones via thiol-ene click reaction. Polym. Int..

[38] Shoda S., Uyama H., Kadokawa J., Kimura S., Kobayashi S. (2016). Enzymes as green catalysts for precision macromolecular synthesis. Chem. Rev..

[39] Gross R.A., Ganesh M., Lu W. (2010). Enzyme-catalysis breathes new life into polyester condensation polymerizations. Trends Biotechnol..

[40] Doukaa A., Vouyioukaa S., Papaspyridi L., Papaspyridesa C.D. (2018). A review on enzymatic polymerization to produce polycondensation polymers: The case of aliphatic polyesters, polyamides and polyesteramides. Prog. Polym. Sci..

[41] Pellis A., Acero E.H., Ferrario V., Ribitsch D., Guebitz G.M., Gardossi L. (2016). The closure of the cycle: enzymatic synthesis and functionalization of bio-based polyesters. Trends Biotechnol..

[42] Jiang Y., Loos K. (2016). Enzymatic synthesis of biobased polyesters and polyamides. Polymers.

[43] Anderson E.M., Larsson K.M., Kirk O. (1998). One biocatalyst-many applications: The use of Candida antarctica. Biocatal. Biotransfor..

[44] Gustini L., Finzel L., Lavilla C., Noordover B.A.J., Hendrix M.M.R.M., Gehrels C., Koning C.E. (2016). Understanding the limitations of the solvent-free enzymatic synthesis of sorbitol-containing polyesters. Acs Sustain. Chem. Eng..

[45] Garcia-Galan C., Berenguer-Murcia Á., Fernandez-Lafuente R., Rodrigues R.C. (2011). Potential of different enzyme immobilization strategies to improve enzyme performance. Adv. Synth. Catal..

[46] Rodrigues R.C., Virgen-Ortíz J.J., dos Santos J.C.S., Berenguer-Murcia Á., Alcantara A.R., Barbosa O., Ortiz C., Fernandez-Lafuente R. (2019). Immobilization of lipases on hydrophobic supports: immobilization mechanism, advantages, problems, and solutions. Biotechnol. Adv..

[47] Ortiz C., Ferreira M.L., Barbosa O., dos Santos J.C.S., Rodrigues R.C., Berenguer-Murcia Á., Briand L.E., Fernandez-Lafuente R. (2019). Novozym 435: the “perfect” lipase immobilized biocatalyst?. Catal. Sci. Technol..

[48] Khan A., Sharma S.K., Kumar A., Watterson A.C., Kumar J., Parmar V.S. (2014). Novozym 435-catalyzed syntheses of polyesters and polyamides of medicinal and industrial relevance. Chem. Sus. Chem..

[49] Skaria S., Smet M., Frey H. (2002). Enzyme-catalyzed synthesis of hyperbranched aliphatic polyesters. Macromol. Rapid Commun..

[50] Kulshrestha A.S., Gao W., Fu H., Gross R.A. (2007). Synthesis and characterization of branched polymers from lipase-catalyzed trimethylolpropane copolymerizations. Biomacromolecules.

[51] Liu C., Jiang Z., Decatur J., Xie W., Gross R.A. (2011). Chain growth and branch structure formation during lipase-catalyzed synthesis of aliphatic polycarbonate polyols. Macromolecules.

[52] Xu F., Zhong J., Qian X., Li Y., Lin X., Wu Q. (2013). Multifunctional poly(amine-ester)-type hyperbranched polymers: Lipase-catalyzed green synthesis, characterization, biocompatibility, drug loading and anticancer activity. Polym. Chem..

[53] Valverde C., Lligadas G., Ronda J.C., Galià M., Cádiz V. (2018). PEG-modified poly(10,11-dihydroxyundecanoic acid) amphiphilic copolymers. Grafting versus macromonomer copolymerization approaches using CALB. Eur. Polym. J..

[54] Wu W. (2019). Lipase-catalyzed synthesis of aliphatic poly(β-thioether ester) with various methylene group contents: thermal properties, crystallization and degradation. Polym. Int..

[55] Wu W., Qu L., Liu B., Zhang W., Wang N., Yu X. (2015). Lipase-catalyzed synthesis of acid-degradable poly (β-thioether ester) and poly (β-thioether ester-*co*-lactone) copolymers. Polymer.

[56] Wu W., Li J., Yang X., Wang N., Yu X. (2019). Lipase-catalyzed synthesis of renewable acid-degradable poly(β-thioether ester) and poly(β-thioether ester-*co*-ricinoleic acid) copolymers derived from castor oil. Eur. Polym. J..

[57] Wu W., Li J., Yang X., Wang X., Zhang Y., Li H., Lan L., Deng J., Wang N., Yu X. (2019). Lipase-catalyzed synthesis of pH-responsive poly(β-thioether ester)-*b*-poly(ethylene glycol)-*b*-poly(β-thioether ester) amphiphilic triblock copolymers for drug delivery. Int. J. Polym. Mater..

[58] Ruiz L., Lligadas G., Ronda J.C., Galià M., Cádiz V. (2019). Synthesis of acid degradable oxidation responsive poly(β-thioether ester)s from castor oil. Eur. Polym. J..

[59] Li L., Wang Q., Lyu R., Yu L., Su S., Du F., Li Z. (2018). Synthesis of a ROS-responsive analogue of poly(*ε*-caprolactone) by living ring-opening polymerization of 1,4-oxathiepan-7-one. Polym. Chem..

[60] Hólter D., Burgath A., Frey H. (1997). Degree of branching in hyperbranched polymers. 3 Copolymerization of ABm-monomers with AB and ABn-monomers. Acta. Polym..

