# Supplementary material for: Novozym 435-Catalyzed Synthesis of Well-Defined Hyperbranched Aliphatic Poly(β-thioether ester)
Source: Molecules. 2020 Feb 6;25(3):687. doi: 10.3390/molecules25030687 (PMC7037349; doi:10.3390/molecules25030687)
Supplement: Supplementary file 1 [file molecules-25-00687-s001.pdf]

Supplementary Information

Novozym 435-catalyzed synthesis of well-defined hyperbranched aliphatic poly(β-thioether ester)

Wan-Xia Wu*, Zi Liu

College of Pharmacy and Biological Engineering, Chengdu University, Chengdu 610106, China

Table of contents for supporting information

Fig. S1. ^{1}H NMR spectra of OTO in $CDCl_3$.

Fig. S2. ^{1}H NMR spectra of HHTP in $CDCl_3$.

Fig. S3. ^{13}C NMR spectra of HHTP in $CDCl_3$.

Fig. S4. ^{1}H NMR spectra of DHTP in $CDCl_3$.

Fig. S5. ^{13}C NMR spectra of DHTP in $CDCl_3$.

Fig. S6. ^{13}C NMR spectra of hPTE-1 in $CDCl_3$.

Fig. S7. ^{13}C NMR spectra of hPTE-1 in $CDCl_3$.

Fig. S8. DSC curves of hPTE and lPTE copolymers at a cooling rate of 10 ℃ min^{-1}.

Fig. S9. Tyndall effect of hPTE and lPTE nanoparticle solution under red laser irradiation.

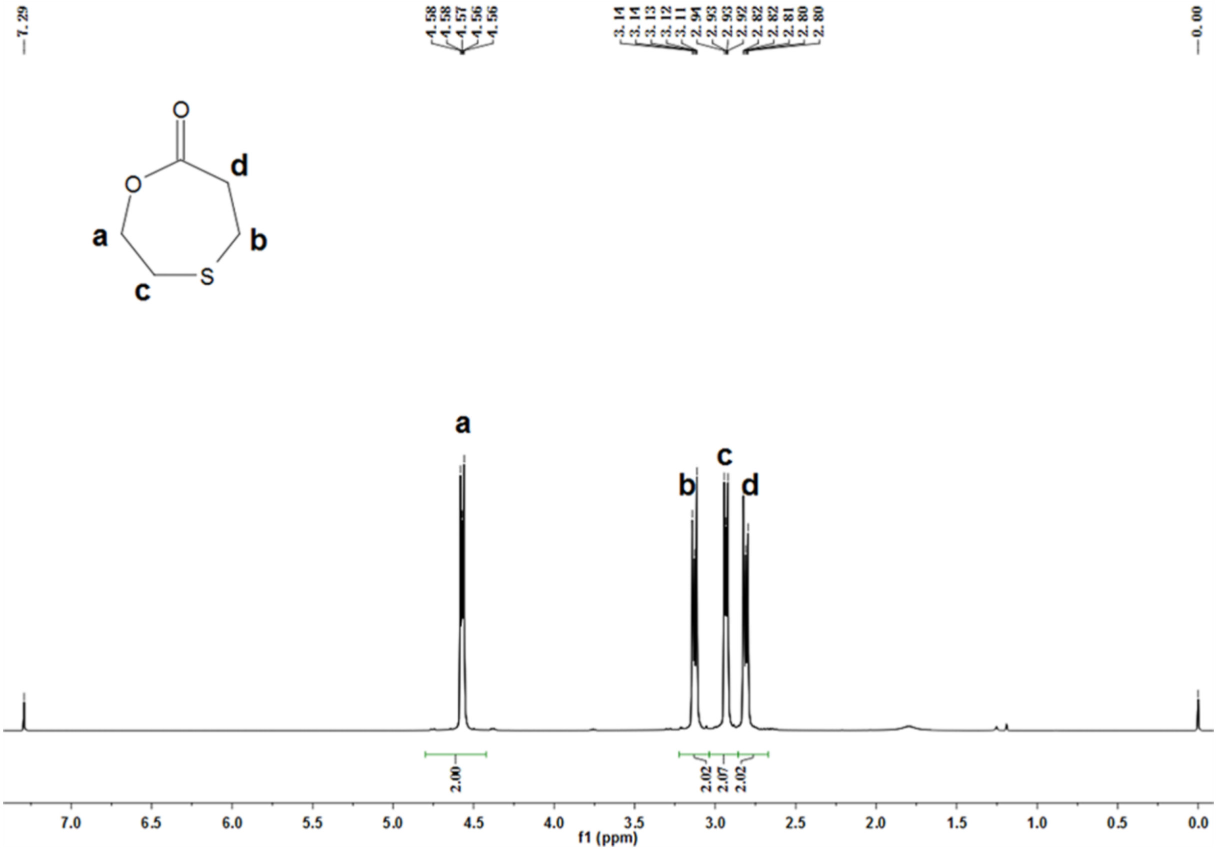

Fig. S1. ^{1}H NMR spectra of OTO in $CDCl_3$.

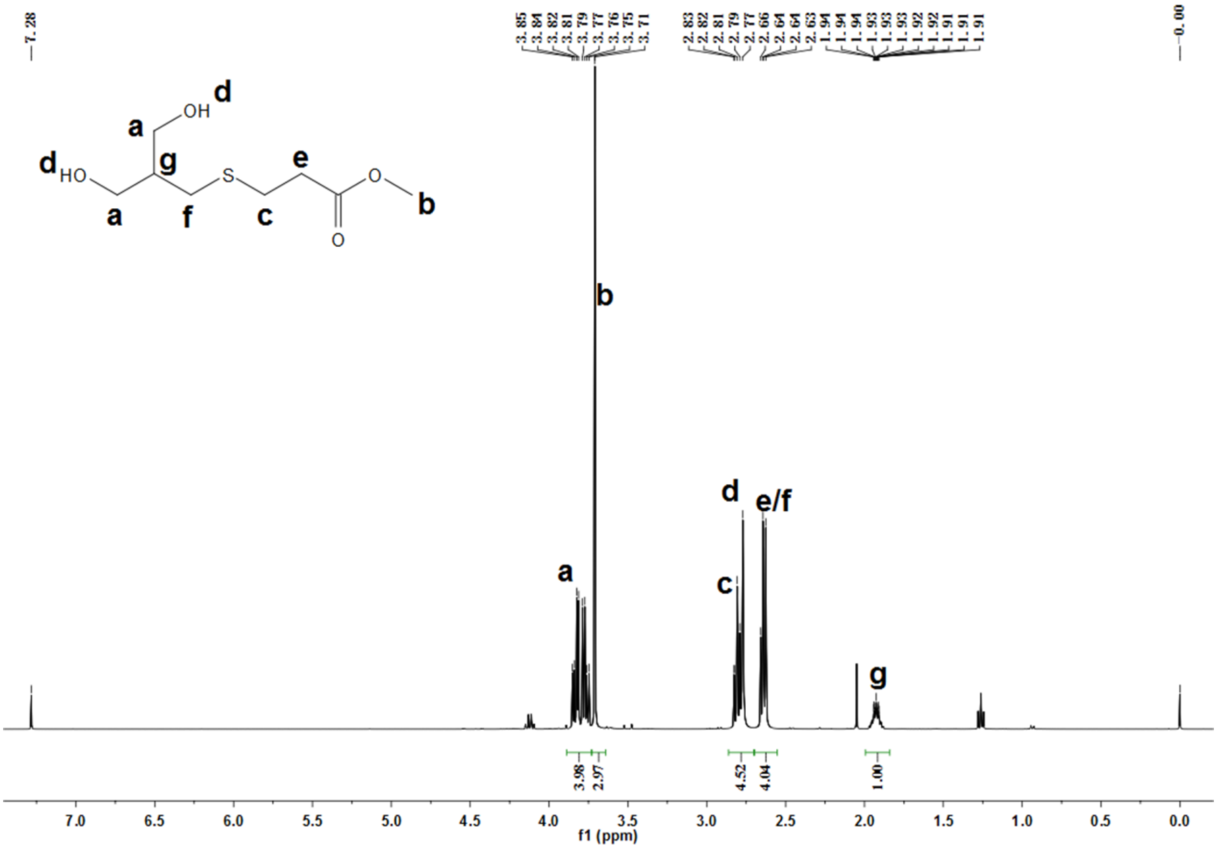

Fig. S2. ^{1}H NMR spectra of HHTP in $CDCl_3$.

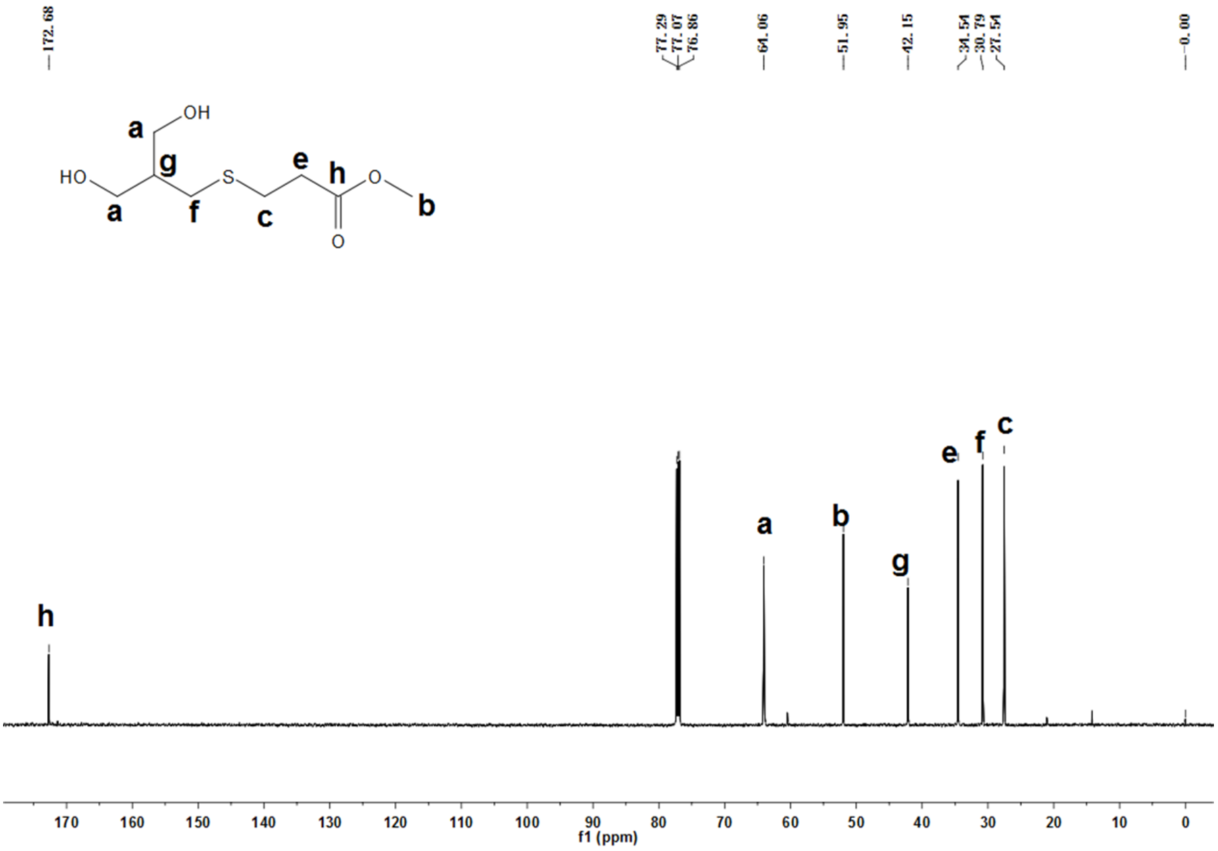

Fig. S3. ^{13}C NMR spectra of HHTP in $CDCl_3$.

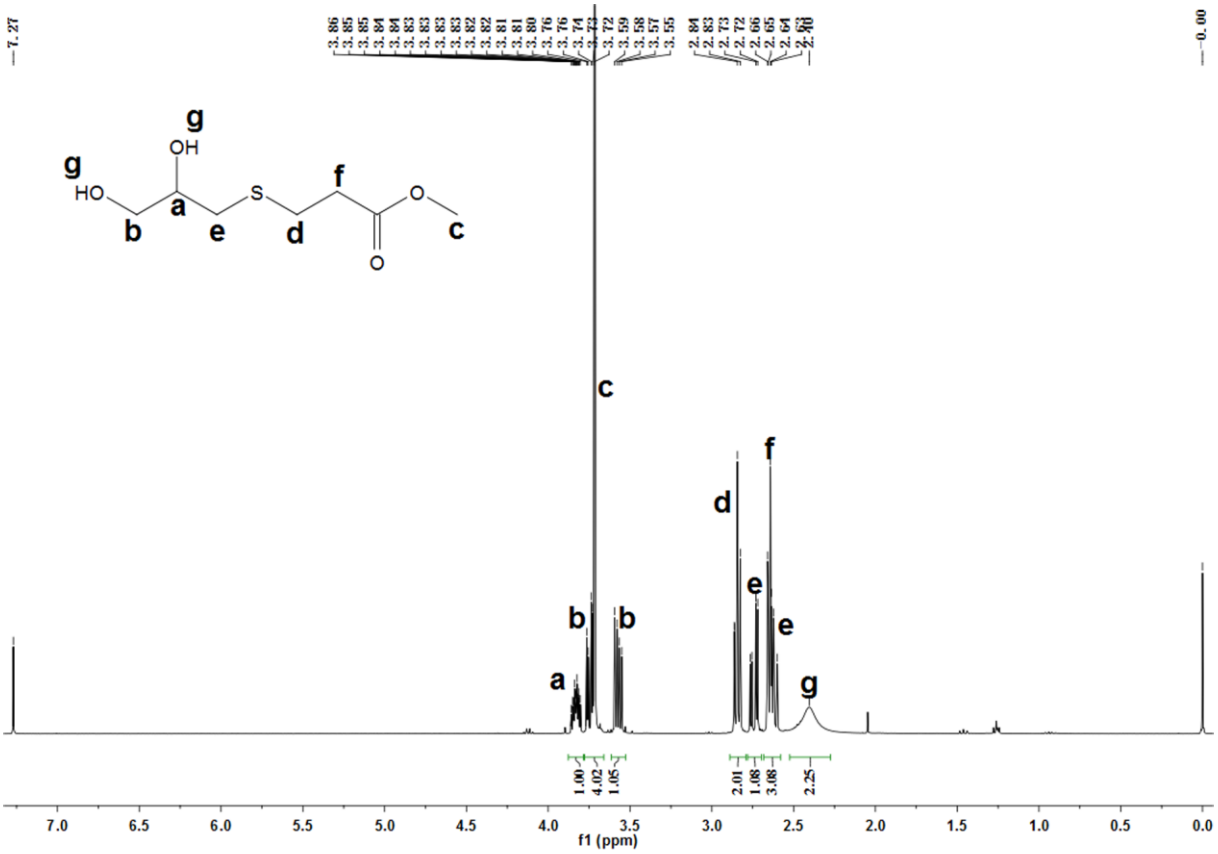

Fig. S4. ^{1}H NMR spectra of DHTP in $CDCl_3$.

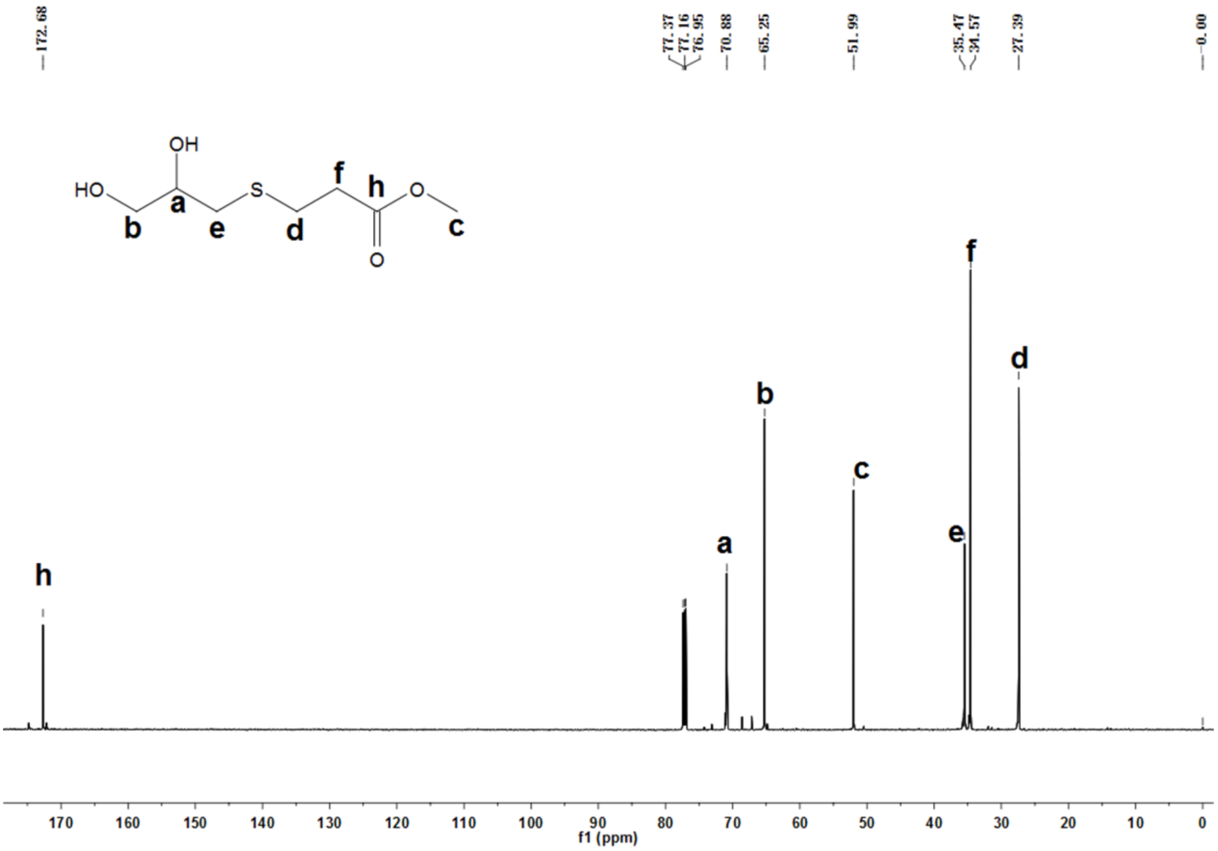

Fig. S5. ^{13}C NMR spectra of DHTP in $CDCl_3$.

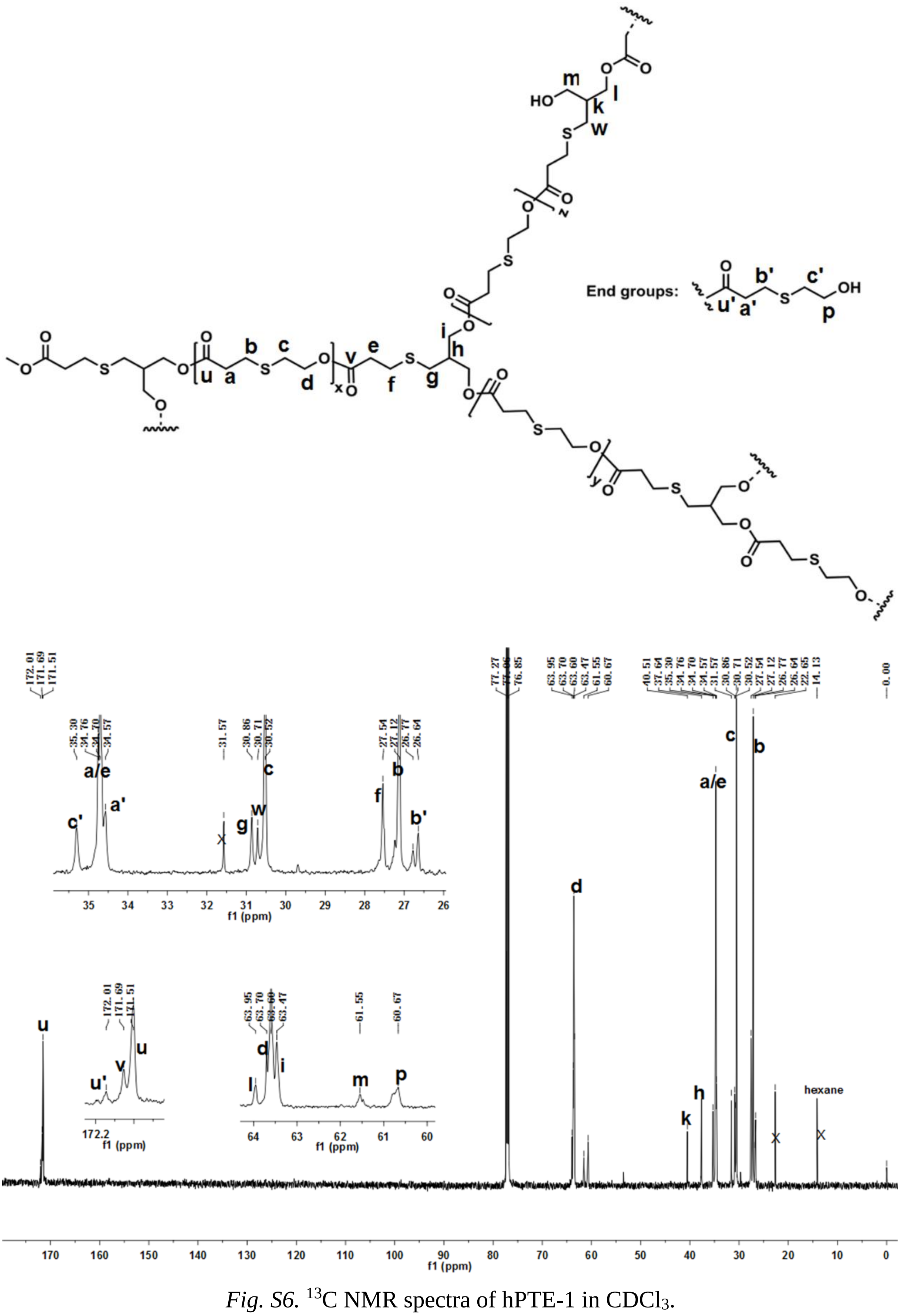

Fig. S6. ^{13}C NMR spectra of hPTE-1 in $CDCl_3$.

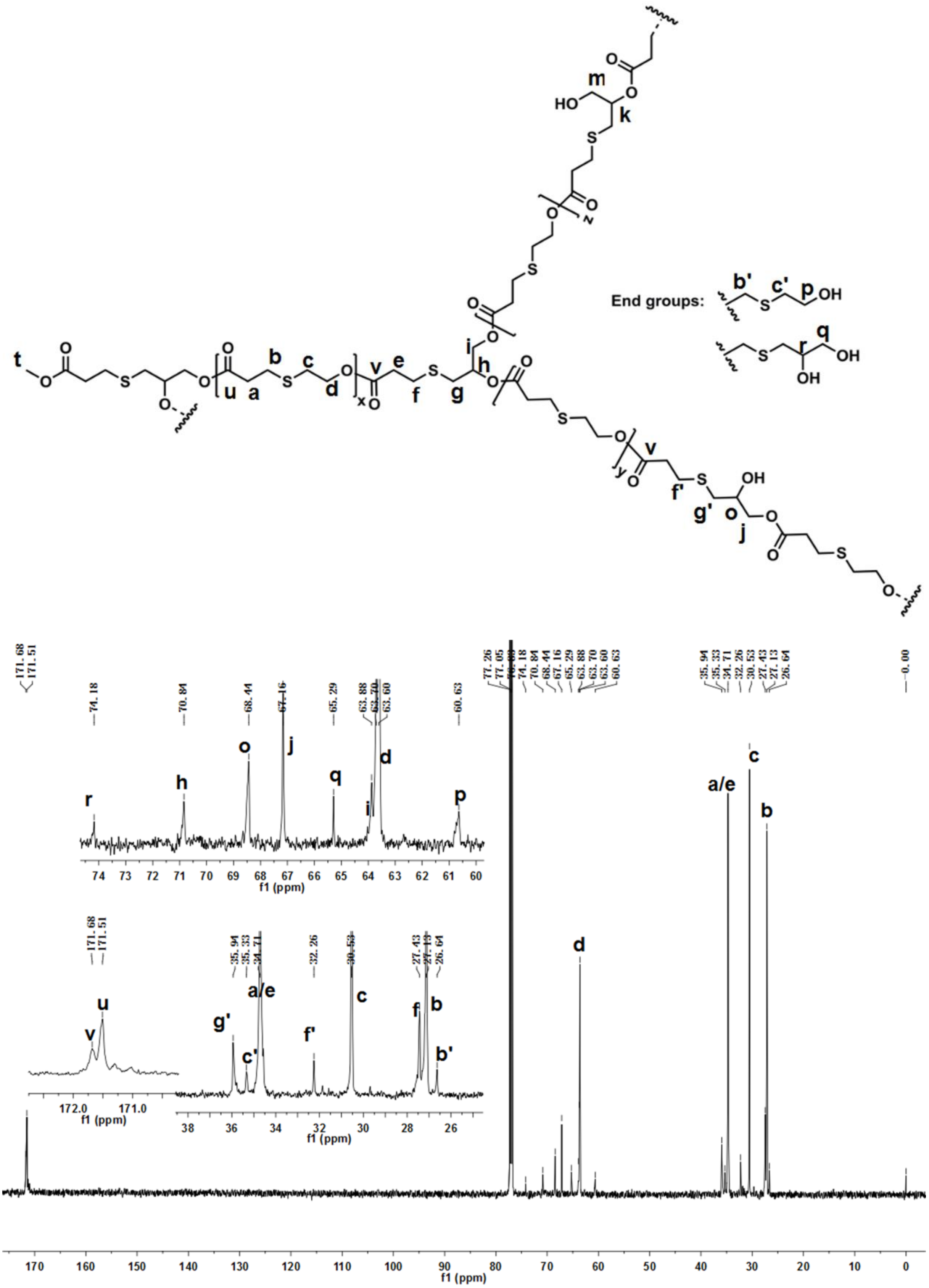

Fig. S7. ^{13}C NMR spectra of lPTE-1in $CDCl_3$.

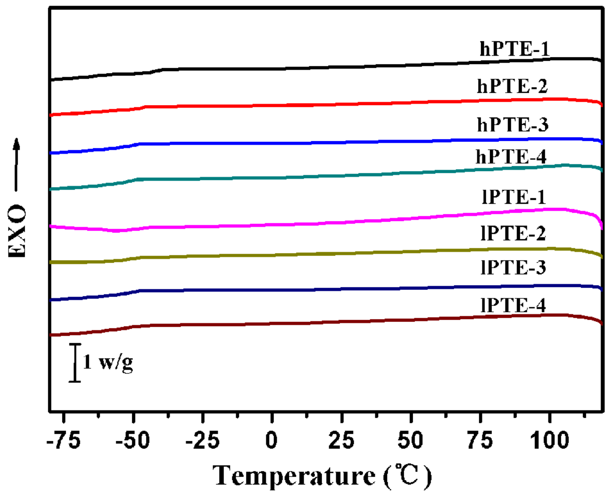

Fig. S8. DSC curves of hPTE and lPTE copolymers at a cooling rate of 10 ℃ min^{-1}.

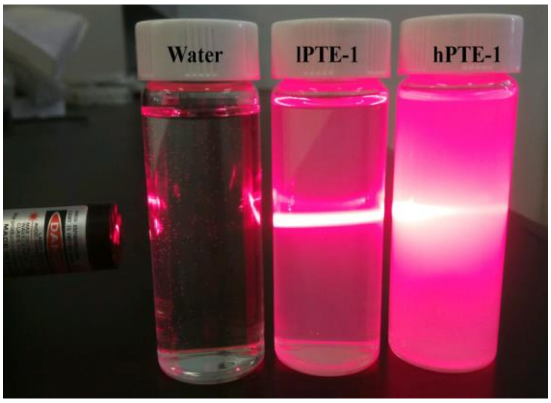

Fig. S9. Tyndall effect of hPTE and lPTE nanoparticle solution under red laser irradiation.
